# The *radical induced cell death* protein 1 (RCD1) supports transcriptional activation of genes for chloroplast antioxidant enzymes

**DOI:** 10.3389/fpls.2014.00475

**Published:** 2014-09-23

**Authors:** Heiko Hiltscher, Radoslaw Rudnik, Jehad Shaikhali, Isabelle Heiber, Marina Mellenthin, Iuri Meirelles Duarte, Günter Schuster, Uwe Kahmann, Margarete Baier

**Affiliations:** ^1^Plant Science, Heinrich-Heine-University DüsseldorfDüsseldorf, Germany; ^2^Plant Physiology, Freie Universität BerlinBerlin, Germany; ^3^Plant Biochemistry and Physiology, Bielefeld UniversityBielefeld, Germany; ^4^Department of Forest Genetics and Plant Physiology, Umea Plant Science Center, Swedish University of Agricultural SciencesUmea, Sweden; ^5^Molecular Cell Physiology, Bielefeld UniversityBielefeld, Germany

**Keywords:** antioxidant system, Arabidopsis, chloroplast, RCD1, ROS

## Abstract

The *rimb1* (*redox imbalanced 1*) mutation was mapped to the RCD1 locus (*radical-induced cell death 1*; At1g32230) demonstrating that a major factor involved in redox-regulation genes for chloroplast antioxidant enzymes and protection against photooxidative stress, RIMB1, is identical to the regulator of disease response reactions and cell death, RCD1. Discovering this link let to our investigation of its regulatory mechanism. We show in yeast that RCD1 can physically interact with the transcription factor Rap2.4a which provides redox-sensitivity to nuclear expression of genes for chloroplast antioxidant enzymes. In the *rimb1* (*rcd1-6*) mutant, a single nucleotide exchange results in a truncated RCD1 protein lacking the transcription factor binding site. Protein-protein interaction between full-length RCD1 and Rap2.4a is supported by H_2_O_2_, but not sensitive to the antioxidants dithiotreitol and ascorbate. In combination with transcript abundance analysis in Arabidopsis, it is concluded that RCD1 stabilizes the Rap2.4-dependent redox-regulation of the genes encoding chloroplast antioxidant enzymes in a widely redox-independent manner. Over the years, *rcd1*-mutant alleles have been described to develop symptoms like chlorosis, lesions along the leaf rims and in the mesophyll and (secondary) induction of extra- and intra-plastidic antioxidant defense mechanisms. All these *rcd1* mutant characteristics were observed in *rcd1-6* to succeed low activation of the chloroplast antioxidant system and glutathione biosynthesis. We conclude that RCD1 protects plant cells from running into reactive oxygen species (ROS)-triggered programs, such as cell death and activation of pathogen-responsive genes (PR genes) and extra-plastidic antioxidant enzymes, by supporting the induction of the chloroplast antioxidant system.

## Introduction

Photosynthesis is accompanied by generation of reactive oxygen species (ROS) which impact on cellular metabolism and trigger signaling cascades (Baier and Dietz, 1999; Samuilov et al., 2002; Bechtold et al., 2008). The chloroplast antioxidant system protects chloroplasts against the destructive potential of ROS (Foyer et al., 1994) and controls spreading of ROS-signals (Zapata et al., 2005). Expression of chloroplast antioxidant enzymes responds dynamically to stressful conditions (Baier et al., 2000, 2004; Kangasjärvi et al., 2008). The signal transduction pathways which adjust it to the chloroplast demands are under investigation.

Most detailed studies on the expressional regulation of the enzymatic components of the chloroplast antioxidant system have been performed for the 2-Cys peroxiredoxin A gene (*2CPA*) of *Arabidopsis thaliana* (Baier et al., 2004; Shaikhali et al., 2008; Shaikhali and Baier, 2010). Peroxiredoxins are an ancient type of peroxidases which detoxify H_2_O_2_ and alkyl hydroperoxides in a thiol-disulfide mechanism (Wood et al., 2003). Regeneration takes place by small thiol proteins, such as thioredoxins and glutaredoxins. *2CPA* transcription activity correlates with the electron pressure at the acceptor site of photosystem-I (Baier et al., 2004), which reflects photosynthetic electron transport intensity relative to acceptor regeneration. Like the other chloroplast antioxidant enzymes, it is nuclear encoded. Transcriptional regulation responds to chloroplast signals (Baier et al., 2004). The signal transduction cascade is independent from the redox state of the plastoquinone pool, ROS and protochlorophyllide signaling, which regulate genes encoding proteins directly involved in photosynthetic electron transport or carbon assimilation, such as *Cab, RbcS* and *PetE* (Pfannschmidt, 2003), and genes for extra-plastidic antioxidant enzymes (Rossel et al., 2007).

The AP2-type transcription factor Rap2.4a was the first identified component of the signal transduction pathway regulating *2CPA* transcription (Shaikhali et al., 2008): Upon slight redox-imbalances Rap2.4a dimerizes. In its dimeric state, it binds the redox box in the *2CPA* promoter and activates *2CPA* transcription. In response to stronger oxidation, it oligomerizes and loses its activation potential on the *2CPA* promoter. Besides *2CPA*, Rap2.4a also orchestrates activation of various other genes encoding chloroplast antioxidant enzymes, such as stromal ascorbate peroxidase (*sAPx*) and copper/zinc superoxide dismutase (*Csd2*) (Shaikhali et al., 2008). In absence of Rap2.4a, *Arabidopsis thaliana* develops photooxidative stress symptoms, such as chlorosis (Shaikhali et al., 2008).

To identify further elements involved in transcriptional regulation of genes for chloroplast antioxidant enzymes, a screen for *redox-imbalanced* mutants, the *rimb*-mutants, was set up in *Arabidopsis thaliana* (Heiber et al., 2007). A reporter gene line expressing luciferase under control of the 2CPA promoter was mutagenized with ethyl methansulfonate and mutants were selected in which the *2CPA* promoter is less activated at the seedling state than in wildtype plants. Only lines were further propagated which were phenotypically indistinguishable from wildtype plants at seedling age.

In the mutant *rimb1, 2CPA* transcription levels are strongest decreased. Also stromal ascorbate peroxidase (*sAPx*), thylakoid ascorbate peroxidase (*tAPx*), CuZn-superoxide dismutase (*Csd2*), peroxiredoxin II E (*PrxIIE*), γ-glutamyl cysteine synthase (*γECS*) and monodehydroascorbate reductase (*MDHAR*) transcript levels are lower than in the non-mutagenized parental line T19-2. Glutathione, ascorbate and chloroplast proteins are more oxidized in the mutant and glutathione biosynthesis is low. Consistent with insufficient chloroplast antioxidant protection, genes for extra-plastidic antioxidant defense enzymes are activated and the mutant develops stress symptoms, such as chlorosis, necrosis and early leaf senescence in the rosette state (Heiber et al., 2007). We concluded that RIMB1 is a major regulator controlling nuclear expression of various chloroplast antioxidant enzymes.

Here, we identified the nature of RIMB1 by map-based cloning. Mapping it to the *rcd1*-locus, which was previously described to protect plants from running into radical induced cell death (Overmyer et al., 2005), protecting from stress-induced senescence (Vainonen et al., 2012) and induction of disease response reactions (Zhu et al., 2013), combined so far independent lines of signal transduction analysis and showed that activation of the chloroplast antioxidant system protects plants from redox-imbalances and running into destructive and cost-intensive ROS-signaling cascades.

## Materials and methods

### Plant material and growth conditions

The reporter gene line T19-2, which expresses luciferase under control of the *2CPA* promoter (Baier et al., 2004), and the *rimb1* mutant (Heiber et al., 2007) were taken from our own seed collection. All other T-DNA-insertion lines were retrieved from the Nottingham Arabidopsis Stock Centre or INRA Versailles. All external lines were tested by PCR for the T-DNA insertion site and homozygosis of the T-DNA insertion with T-DNA-flanking primers according to standard PCR protocols.

The plants were grown on soil as described previously (Heiber et al., 2007; Juszczak et al., 2012). Aseptic growth on plates was performed as described in Baier et al. (2004). Our standard conditions in the growth chambers were: 10 h light (80–100 μmol quanta m^−2^ s^−1^) at 20°C and 14 h darkness at 18°C and all day between 50 and 60% humidity. In the greenhouses the conditions were more variable as described in Suppl. 1.

For paraquat treatment, 2 week old aseptically grown plants were sprayed with 1.5 μM paraquat. Seven days later, the survival rates were determined by comparison of the number of vital/green plants with the number of total plants.

### Luciferase activity measurements and mapping

Plant populations were scored for luciferase activity levels as reported in Heiber et al. (2007). Mapping was performed with SSLP and CAPS markers and DNA isolated according to standard procedures (Jander et al., 2002). Fragments of 180–300 bp were amplified, (in case of CAPS markers digested) and separated on 4 % (w/v) TAE-agarose gels containing ethidium bromide and analyzed fluormetrically according to standard procedures.

The *Rcd1* gene was amplified from genomic DNA with gene specific primers by PCR using a proof-reading polymerase. The amplificates were cloned into pJET1.2 (Fermentas, St.-Leon-Rot, Germany) following blunting of the DNA ends. The plasmids were transformed in OneShot TOPO cells for amplification, re-isolated and purified with the Wizard Plus Minipreps DNA Purification System by Promega (Munich, Germany) and sequenced by GATC Biotech (Konstanz, Germany).

For allelism testing, the mutant was crossed to the non-mutagenized background line T19-2 (Heiber et al., 2007) and to *rcd1-3*. In crosses A × B, the mother plant is named first (here: A) and the name of the pollinator second (here: B).

### RNA isolation, cDNA synthesis, and RT-PCR

RNA isolation, cDNA synthesis and RT-PCR were performed according to Baier et al. (2000) with gene specific primers amplifying the 5′-end of the cDNAs (Suppl. 2) or with QuantPrime-optimized primers (Arvidsson et al., 2008) spanning exon-intron borders (Suppl. 3) according to the MIQE standards, as described previously in Juszczak et al. (2012), using actin cDNAs as references.

### Determination of habitus parameters

Habitus parameters, such as root lengths and root branching, were determined after digital imaging using the software packages EZ-Rhizo (Armengaud et al., 2009) and ImageJ (Sheffield, 2007).

### Light microscopy

Cell size parameters and stomata densities were determined with fresh epidermis stripes using a Zeiss Axio Imager Light microscope and the Axio vision (Zeiss, Jena, Germany) and ImageJ (Sheffield, 2007) software packages.

### Electron microscopy

For scanning electron microscopy, freshly cut leaf sections were transferred to brazen specimen holders and gold-sputter coated for 180 s at 35 mA using a Agar Sputter Coater (Agar Scientific Ltd., UK) and analyzed by scanning electron microscopy [SEM; LEO 1430 (VP), LEO Ltd., Germany] at 20–21 kV and a working distance of 30 mm. Scaling was automatically performed using the intrinsic calibration mode. Transmission electron microscopy was performed as described in König et al. (2002).

### Yeast-2-hybrid protein-protein interaction tests

The *rcd1* cDNAs were amplified from wildtype and mutant material using the primers AAAAAGAATTCATGGAAGCCAAGATCGTC, and AAAACCCGGTTACAATCCACCTGCACC. The amplificates were cloned into the EcoRI/SmaI-sites of pGBT9 (Clontech Laboratories, Mountain View, USA) upstream of the *HIS3* gene. The yeast strain Y187 was co-transformed with pGBT9-RCD1 and pACT2-clone1 (Shaikhali et al., 2008) encoding Rap2.4a. The transformants were grown for 6 days at 30°C on SD media lacking tryptophan and leucine (Ausubel et al., 2001). Positive interactions were tested in β-galactosidase colony-lift filter assays as described in Schneider et al. (1996).

Alternatively interactions were tested in the HF7c yeast strain co-transformed with pGBT9-RCD1 and pACT2-clone1 (Shaikhali et al., 2008) on SD media lacking histidine, tryptophan and leucine, and supplemented with 20 mM 3-amino-1,2,4-triazole (3-AT).

For effector treatments, pGBT9-RCD1 + pACT2-clone1 double transformed Y187 cells were grown in liquid SD media lacking tryptophan and leucine. Aliquots were supplemented with H_2_O_2_, ascorbate and DTT. As positive control, commercially available pVA and pTD1 plasmids were co-transformed for co-expression of the strongly interacting proteins p53 and SV 40 large T-Antigen (Li et al., 1994).

### Chlorophyll-a fluorescence analysis

Chlorophyll-fluorescence was analyzed with a MINI-PAM fluorimeter (Walz, Effeltrich, Germany) after 30 min dark treatment. The maximum quantum yield (F_V_/F_M_) was determined with a saturating light flash (>2000 μmol quanta m^−2^ s^−1^). Afterwards the plants were illuminated for 5 min with actinic light (180 μmol quanta m^−2^ s^−1^). The efficient quantum yield of photosystem II (F_V′_/F_M′_) and photochemical quenching were determined every 20 s with a saturating light flash. Calculations were performed as described in Schreiber and Bilger (1993).

### Statistical analysis

Significance of difference was analyzed by Student's *T*-Test and the independence by X^2^-testing. For the latter, the results observed with the non-mutagenized background line T19-2 (Baier et al., 2004) were defined as expectation.

## Results

### Phenotype-based selection of a *rimb1*-positive mapping population

The *rimb* mutants were generated by mutagenizing the reporter gene line T19-2, which expresses luciferase under control of the *2CPA*-promoter (Heiber et al., 2007). For mapping of the recessive mutant locus, the segregating F2 population of the cross of *rimb1* to the *Arabidopsis* wildtype *Landsberg erecta* (Ler) was screened for low luciferase activity. Applying the same scoring procedure as previously used with segregating backcross populations (*rimb1* × T19-2 and T19-2 × *rimb1*) (Heiber et al., 2007), almost twice the expected number of plants (47.3% of the F2 seedlings, genotyped to be positive for the reporter gene construct by PCR) showed low luciferase activity.

To separate the *rimb1* mutant locus from the interfering accession-specific locus, F2 lines with less than 60% luciferase activity (relative to the parental line) were sub-classified into 4 groups according to their luciferase activity level. The F3-progenies were grown side by side in the greenhouse and monitored for growth and color phenotypes. In three independently grown sub-populations of at least 200 plants with 25–30% luciferase at an age of 10 days, between 50 and 83% of the plants developed chlorotic and necrotic lesions in the rosette state, as previously observed for *rimb1* (Heiber et al., 2007). Later during development, necrosis, limited leaf blade growth and wrinkling of the leaf surface were observed. In contrast, chlorosis and abnormal leaf development were only observed in up to 6% of the plants in sub-populations with >60% of luciferase activity (relative to the parental line T19-2) or in plants with less than 15% luciferase activity indicating linkage of the leaf phenotype and medium-low-luciferase phenotype.

### Co-segregation of low-luciferase with abnormal, chlorotic leaf development

**Figure 1:**
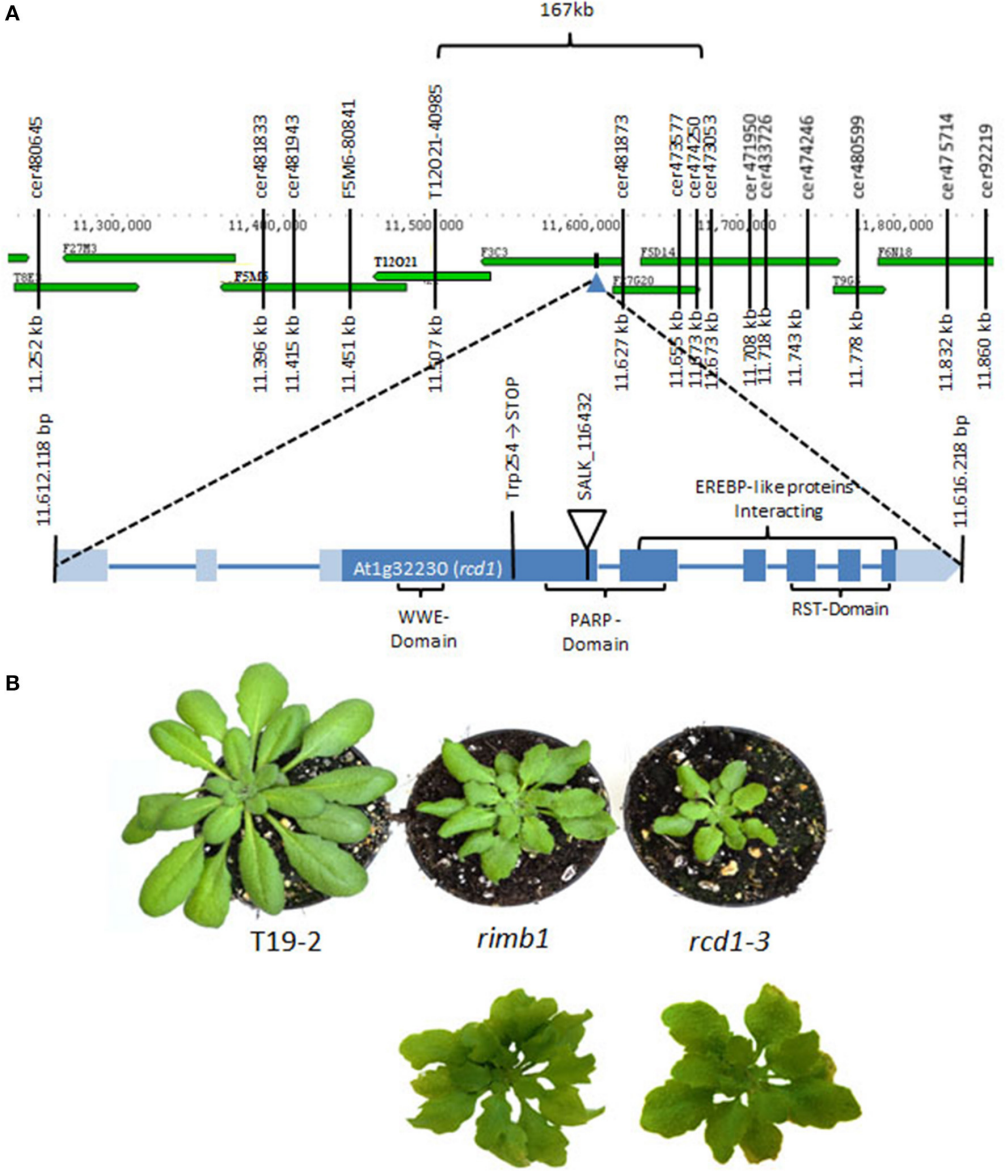
**Mapping of the *rimb1* mutation**. **(A)** The *rimb1* mutation was mapped to a 167 kb region on chromosome 1 with SSLP and CAPS markers. Following analysis of the target region with T-DNA insertion mutants, the mutation (G→A) was identified in the Trp254 codon of gene At1g32230 by sequencing. **(B)** Top: Comparison of *rimb1* mutants with the RCD1-deficient SALK-line_11643 (*rcd1-3*) (average phenotypes) after 6 weeks at short-day conditions. Bottom: The most severe leaf form phenotypes in parallel grown *rimb1* and *rcd1-3* populations.

To proof that abnormal leaf development in the rosette stage co-segregates with low luciferase activity at seedling age, phenotypically normal (similar to T19-2; Figure 1B) and abnormal plants (similar to *rimb1* (*rcd1-6*); Figure 1B) were selected from a population of 300 soil-grown F2 plants of the cross of *rimb1* and Ler (without pre-screening for luciferase activity). The F3 progeny of 20 plants with clearly abnormal leaves (chlorotic, serrated and/or necrotic) and 40 plants with clearly normally shaped and green leaves were screened for luciferase activity at 10 days, when the mutant seedlings were still visually indistinguishable from wildtype (Heiber et al., 2007). For all 20 lines with leaf defects in the F2 generation, the *2CPA*-promoter-driven luciferase activity was decreased in 10 day old F3 seedlings.

From the lines, which showed a wildtype-like habitus in the F2 generation in the rosette state, in 31 lines the luciferase activity was not decreased in F3 generation. In the 9 lines with decreased luciferase activity of (by average) 56% of the F3 plants developed abnormally shaped leaves at an age of 6 weeks. In parallel grown plants of the non-mutagenized parental line T19-2, only up to 2% of the F3 plants developed chlorosis and necrosis at an age of 6–8 weeks. According to X^2^-testing, the numbers showed that low luciferase activity correlates with the phenotypical difference to the parental line (error probability: <10^−15^). For all 20 abnormal lines the *2CPA*-promoter-driven luciferase activity was decreased demonstrating co-segregation of a low luciferase activity level with the phenotype (error probability: <10^−40^).

### Mapping of the *rimb1*-mutant locus

Due to the correlation of abnormal leaf development with low luciferase activity, the *rimb1*-mapping population was extended by screening the F2 progeny of *rimb1*xLer for the leaf phenotype. 652 lines with lighter green and abnormally shaped leaves were selected. The mapping population was purified by excluding all lines, which had lost the luciferase construct in the F2 (according to PCR-based genotyping with primers binding the luciferase-cDNA) and rescreened in the F3 for low luciferase activity. With 350 luciferase-positive lines the mutation was mapped with SSLP and CAPS markers to a 167 kb region on chromosome 1 (Figure 1A). The target region between the markers Cer474250 and T12O21 was screened for candidate genes by comparing the habitus of T-DNA insertion lines with *rimb1*.

The homozygous offspring of SALK-Line_116432 (*rcd1-3*) (Katiyar-Agarwal et al., 2006) was phenotypically similar to *rimb1* (Figure 1B). *rcd1-3* carries a T-DNA insertion in gene At1g32230 encoding RADICAL INDUCED CELL DEATH-1 (RCD1) (Teotia and Lamb, 2009; Figure 1A).

**Figure 2:**
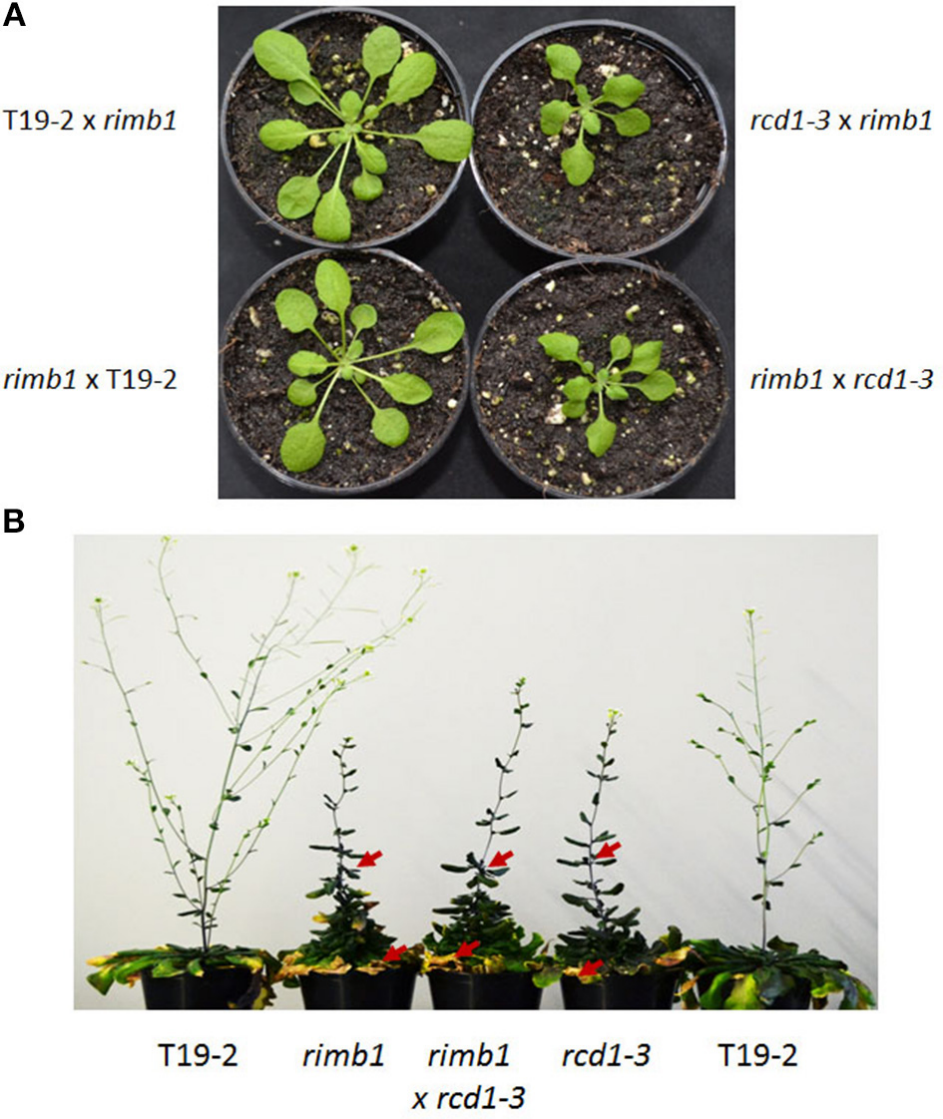
**Allelism test. (A)** Habitus of 5 week old F1 plants of the cross of *rimb1* (*rcd1-6*) to T19-2 and *rcd1-3* and F1 plants of crosses of T19-2 and *rcd1-3* to *rimb1* (*rcd1-6*) grown under short-day conditions. Representative plants from populations of 45–51 F1 plants are shown. **(B)** Habitus of flowering T19-2 plants, *rimb1* (*rcd1-6*) and *rcd1-3* and F1 plants of the cross of *rcd1-3* to *rimb1* (*rcd1-6*) grown under short-day conditions. The red arrows mark aerial rosettes and necrotic leaves. Representative plants from parallel grown populations of 50 (F1 of the cross *rimb1xrcd1-3*) and 18–20 T19-2, *rimb1* and *rcd1-3* plants are shown.

*rimb1* and *rcd1-3* showed lighter green and stronger serrated leaves under short day conditions in controlled environment (10 h 100 μmol quanta m^−2^ s^−1^; 20°C; 14 h dark at 18°C) (Figures 1B, 2). Later in both lines, small rosettes with 3–5 leaves were formed at the knots along the inflorescence axis instead of cauline leaves in both lines (Figure 2B) indicating that *rimb1* is allelic to *rcd1* (Ahlfors et al., 2004).

### Genetic allelism test

Allelism was tested by crossing the recessive *rimb1* mutant (Heiber et al., 2007) to the *rcd1-3* T-DNA insertion line and *rcd1-3* to *rimb1*. In the F1 generation, 64 and 67% of the plants showed the *rimb1*- and *rcd1-3*-typical abnormal leaf habitus, while none of the F1 progeny of crosses of *rimb1* to T19-2 and T19-2 to *rimb1* did (Figure 2). The number of phenotypically positive F1 plants of the crosses *rimb1xrcd1-3* or *rcd1-3xrimb1* was in the range of the penetrance of the phenotype in parallel grown homozygous *rimb1* and *rcd1-3* plants (60 and 78%) and the average phenotype variations (Suppl. 1) demonstrating that *rimb1* is an *rcd1* allele and, therefore, that the low activation of genes for chloroplast antioxidant enzymes (Heiber et al., 2007) results from a defect in the same gene, which protect Arabidopsis from running into cell death (Overmyer et al., 2005).

### Sequencing-based identification of the mutation

For the final proof of identity, the *rcd1* gene was amplified by PCR from genomic DNA of *rimb1* and the parental line T19-2. The PCR products were cloned, sequenced and compared. In the *rcd1*-DNA sequence of *rimb1*, but not in that of T19-2, a single base pair exchange was observed. Exchange of G into A modified the Trp-codon (TGG) at position 254 into a stop codon (TAG) (Figure 1A). *Rimb1* is an *rcd1* allele and was, therefore, renamed as *rcd1-6*.

### Analysis of *rimb1* for physiological and morphological *rcd1* characteristics

**Figure 3:**
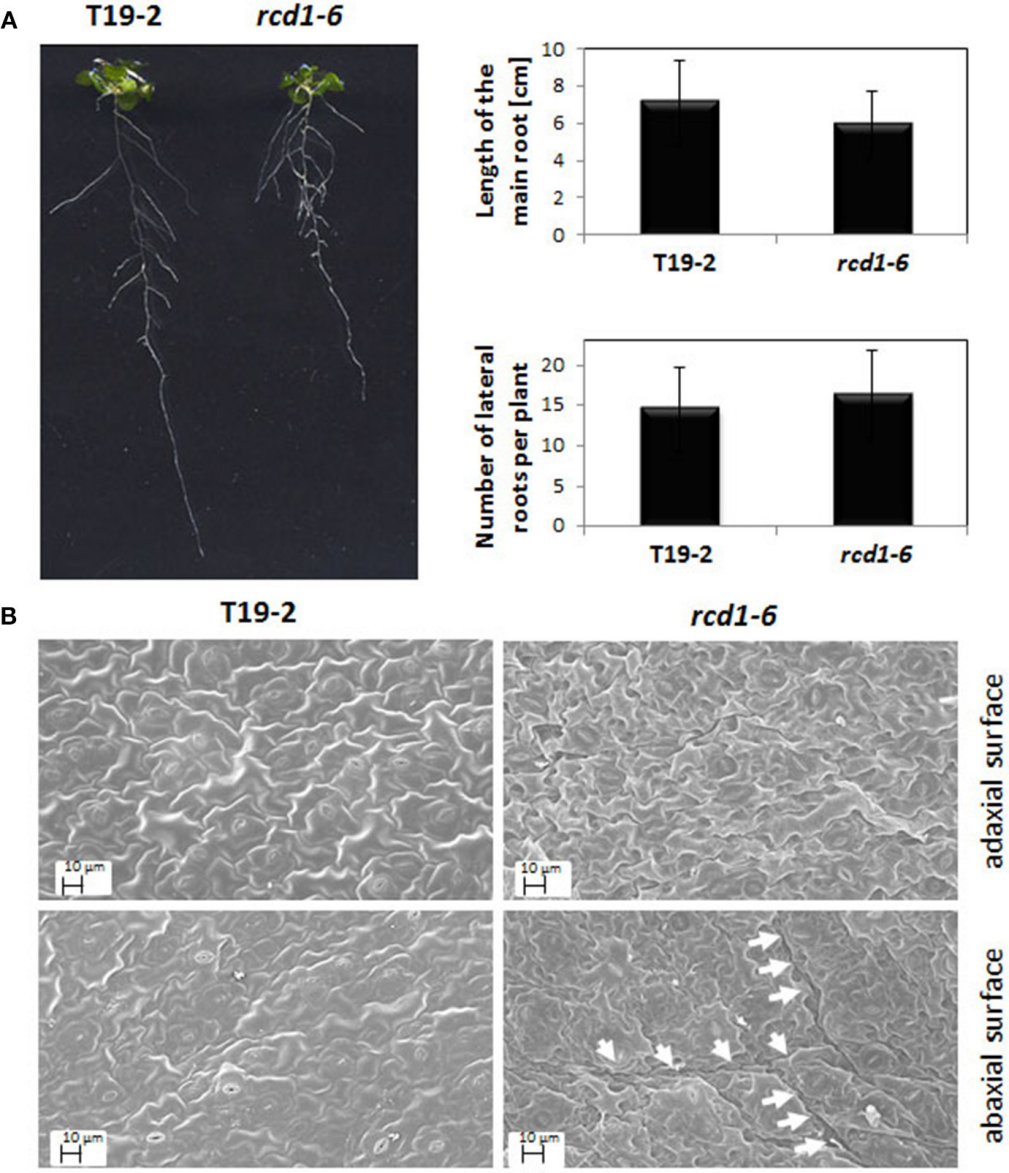
**(A)** Root length and root branching in *rcd1-6 (rimb1)* and the parental line T19-2. Quantitative data were calculated from 30 plants grown aseptically on 0.5 × MS-medium supplemented with 0.5% (w/v) sucrose. The root lengths and the branching intensity were quantified by analysis of digital images with EZ-Rhizo and ImageJ. The length variations and the lateral root numbers were not significantly different as analyzed by Student's *T*-Test (α = 0.1). The photo shows representative T19-2 and *rcd1-6* plants of the analyzed populations. **(B)** Representative scanning electron images of the abaxial and adaxial the surfaces of full-grown leaves of *rcd1-6* (*rimb1)* and the parental line T19-2 grown for 6 weeks in short day (10 h at 80–100 μmol quanta m^−2^ s^−1^/14 h darkness) and a relative humidity of 50–60%. Prior to microscopy the surfaces were sputtered with a thin layer of gold as described in material and methods. The arrows mark the rims of “clefts.”

To describe the *rcd1-6* allele and enable comparison with other *rcd1*-mutants, *rcd1-6* was tested for well-described *rcd1* phenotypes. *Rcd1* mutants were characterized for their decreased sensitivity toward paraquat (Ahlfors et al., 2004; Fujibe et al., 2004). The same was observed for *rcd1-6* (Table 1). *rcd1-6* plants also showed decreased root lengths, but, by average, much milder as reported for *rcd1-3* by Teotia and Lamb (2011). The number of lateral roots was also only slightly increased (Figure 3A right). In highly variable populations, some of the *rcd1-6* mutants showed as strong phenotypes as Teotia and Lamb (2011) reported (Figure 3A left), while T19-2 plants did not.

**Table 1:** **Paraquat resistance of *rcd1* and T19-2 seedlings**.

	***T19-2***	***rimb1 (rcd1-6)***	***rcd1-3***
Lethal plants	211	72	41
Vital plants	6	214	116
Number of plants	217	286	157
Survival rate [%]	2.8	74.8	73.9

*Dead (pale) and vital (green) plants were counted 5 days after spraying of 2 week old plants with 1.5 μM paraquat*.

*Rcd1-6* plants formed by average less than 40% of the flowers wildtype *Arabidopsis* does (Figure 2). The rosette leaves were rougher (Figure 3B). The number of stomata per mm^2^ was with 12.1 ± 5.3 on the upper and 15.56 ± 2.28 on the lower surface decreased compared to T19-2 (20.2 ± 5.6 and 17.6 ± 3.1, respectively). The outer cell surface of the upper epidermis is with 879.4 μm^2^ slightly larger in *rcd1-6* (*rimb1*) than in T19-2.

The cell shape and surface texture was stronger affected in the lower epidermis. Clefts and tissue deformations were observed (Figure 3B) similar to the defects reported by Teotia and Lamb (2011) for *rcd1-3* demonstrating that in *rcd1-6* also groups of neighboring mesophyll cells run into cell death.

### RCD1 can bind Rap2.4a

The *rcd1-6* (*rimb1)* mutant has been isolated for decreased expression of *2CPA* and other genes for chloroplast antioxidant enzymes at seedling stage (Heiber et al., 2007). The early effect on induction of the chloroplast antioxidant system poses the question how RCD1 is involved in the regulation of nuclear expression of the chloroplast antioxidant enzymes. Previous work (Belles-Boix et al., 2000; Ahlfors et al., 2004; Vainonen et al., 2012) suggested that RCD1 (previously designated CEO1) acts as a scaffold for various transcription factors in order to balance signal transduction cascades (Overmyer et al., 2000). Specificity is provided by distinct protein-protein interaction sites. The C-terminal RST-domain of RCD1 (Jaspers et al., 2010) selects target transcription factors by distinct recognition motifs (Vainonen et al., 2012).

Redox-regulation of *2CPA* is mediated by the AP2-type transcription factor Rap2.4a (Shaikhali et al., 2008). To test whether RCD1 can interact with Rap2.4a under similar conditions (as used by Belles-Boix et al., 2000 to study RCD1-transcription factor interactions), a yeast-two-hybrid approach was chosen. It enables detection of protein-protein-interactions at low expression levels and quantification of the interaction strength in a complex protein environment of a living cell, but avoids problems, which are accompanied with e.g. pull-down assays, if the interaction of a multiple transcription factor binding protein as RCD1 (Belles-Boix et al., 2000; Jaspers et al., 2009, 2010) and a specific (weakly expressed) transcription factor should be studied.

**Figure 4:**
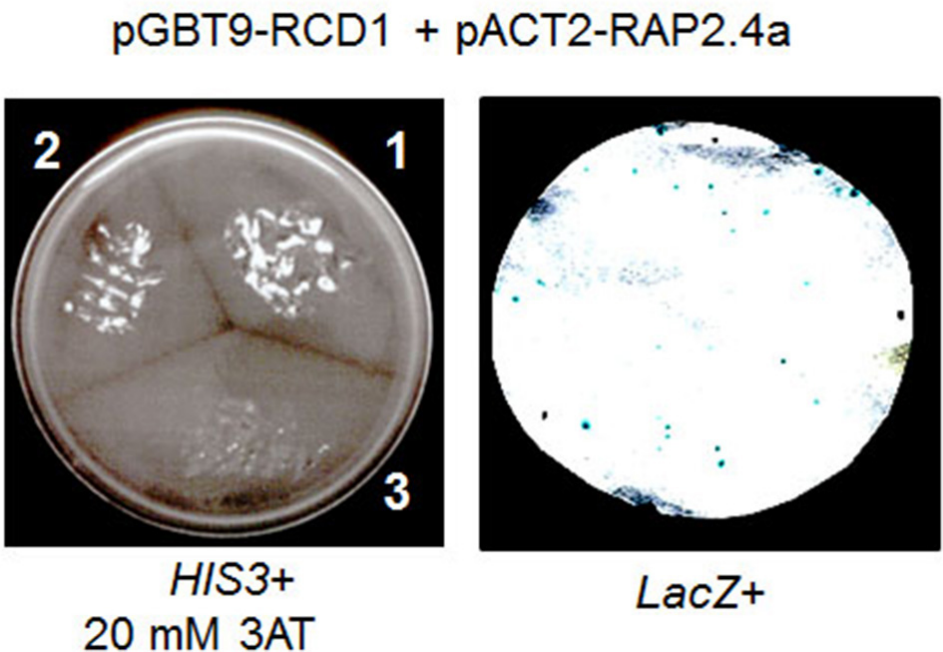
**RCD1-Rap2.4a interaction test in yeast**. His3 activation by interaction of Rap2.4a with RCD1 (segment 2 in the left panel) is compared to the known interaction of pVA3 and pTD1 (segment 1 in the left panel) and a non-transformed control (segment 3 in the left panel). RCD1-Rap2.4 interaction-based activation of the GAL4 promoter was confirmed by monitoring LacZ reporter activation in pGBT9-RCD1 and pAct2-Rap2.4a double transformed yeast cells (right).

The cDNA encoding RCD1 was cloned into the bait vector pGBT9 and the Rap2.4a cDNA into the prey vector pACT2. Double transformants of the yeast strain HF7c showed protein-protein interaction by complementation of the histidine auxotrophy of the yeast strain. On 20 mM 3-AT, which increases the stringency by inhibiting histidine biosynthesis, the double transformant grew almost as good as the commonly used positive control, pVA-pTD1 double transformants (Figure 4 left) (Li et al., 1994). The pAct2-Rap2.4a-empty pGBT9 double transformants (negative control) did not grow.

Additional support for an interaction of Rap2.4a with RCD1 was given by β-galactosidase filter lift assays with double transformed yeast Y187 based on activation of a GAL4-promotor-controlled LacZ reporter gene activation (Figure 4 right). The pGBT9-RCD1 and the pACT-Rap2.4a single transformants of Y187 (autoactivation test) showed no activation of the reporter gene (data not shown).

### The protein-protein interaction of RCD1 and Rap2.4a is only slightly redox-regulated

**Figure 5:**
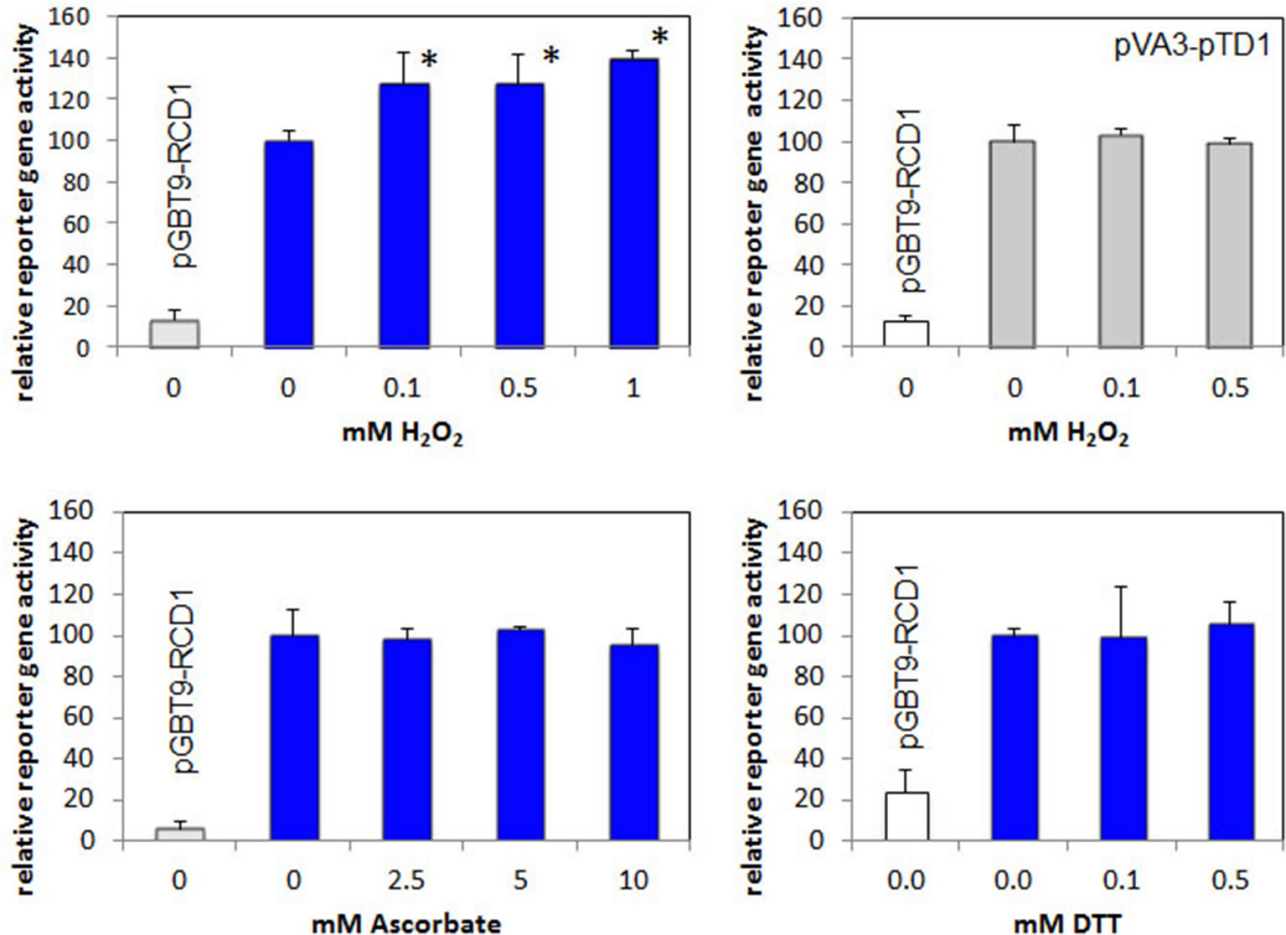
**Analysis of redox regulation of the RCD1–Rap2.4 interaction in yeast by H_2_O_2_, ascorbate and DTT compared to the H_2_O_2_-regulation of pVA3-pTD1**. As a measure for the protein-protein interaction the Gal4-driven LacZ activity was quantified. In all experiments, yeast only transformed with the RCD1-expression construct pGBT-RCD1 served as a negative control. Significant differences to the untreated control (Student's *T*-Test; α = 0.01; *n* = 7) are labeled with an asterisk.

Rap2.4a confers redox-dependent modulation of quaternary structure (Shaikhali et al., 2008). It dimerizes upon slight oxidative shifts and oligomerizes upon severe stress conditions. These structural changes may affect the interaction with other proteins. To test whether the interaction of RCD1 and Rap2.4a is redox-regulated, the interaction specific β-galactosidase activity was used as a quantitative measure to compare the interaction strength. Double transformed yeast strains expressing a RCD1 bait construct (pGBT9-RCD1) and a Rap2.4a prey construct (pACT2-Rap2.4a) were treated with 0–1 mM of the oxidant H_2_O_2_, 0–10 mM of the antioxidant ascorbate and 0–0.5 mM of the reducing disulphide DTT (Figure 5). While DTT and ascorbate did not affect the interaction, a slight increase in the Rap2.4a-RCD1 interaction strength was observed with H_2_O_2_. No H_2_O_2_ response was observed for protein-protein interaction of VA3 and TD1 (encoding SV40 large T-antigen and GAL4-AD and murine p53 protein and GAL4-BD; Li and Fields, 1993) (Figure 5), which served as a negative control.

### Redox-regulation of *RCD1* expression

**Figure 6:**
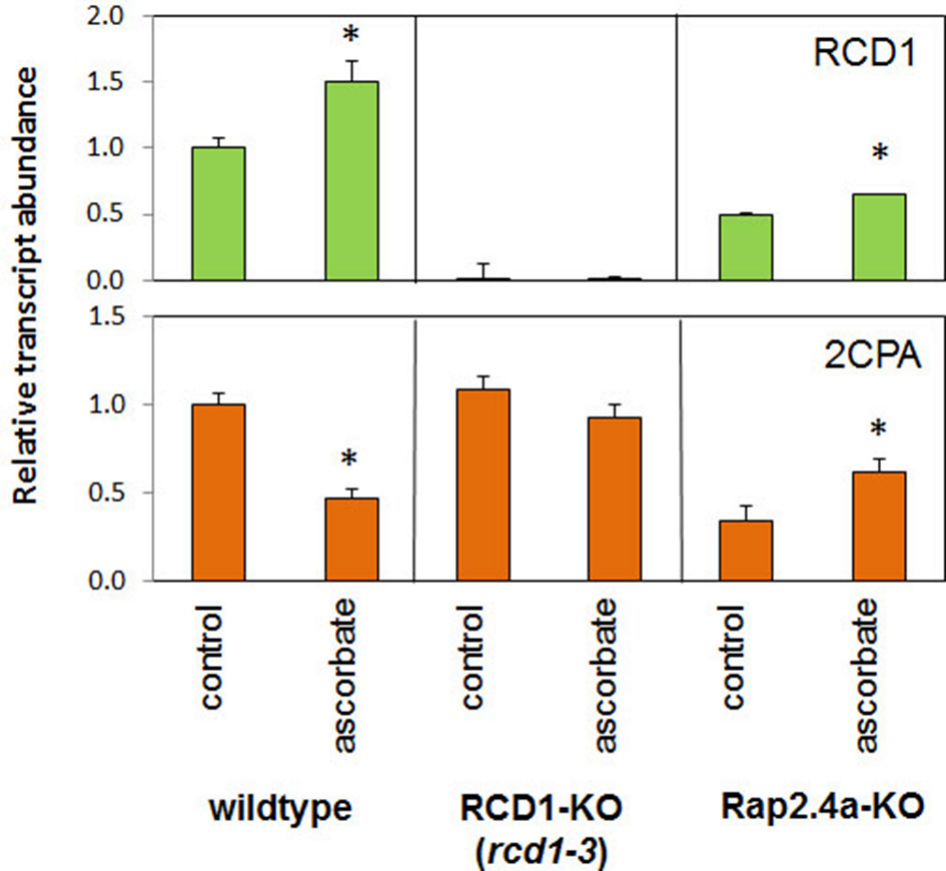
**Transcript abundances of *2CPA* and *RCD1* in 6 week old Arabidopsis wildtype plants and RCD1 and Rap2.4a knock-out lines standardized on actin transcript levels and normalized on the transcript level of the respective gene in wildtype plants under control conditions in response to ascorbate as determined by RT-PCR and gel electrophoresis**. Significant differences to the untreated control (Student's *T*-Test; α = 0.01; *n* = 3) are labeled with an asterisk.

*2CPA* expression is strongly reduced by ascorbate application (Horling et al., 2003; Baier et al., 2004; Figure 6). The ascorbate-controlled transcriptional regulation is light-dependent and, as co-application of DCMU demonstrated, interwoven with the control of *2CPA* expression by photosynthetic signals (Shaikhali and Baier, 2010). To test whether RCD1 is involved in the ascorbate response, *2CPA* and *RCD1* expression was analyzed in *rcd1-3* lines and compared with wildtype and, for further inside into the signaling network with, Rap2.4a knock-out lines in 6 week old plants grown under short-day conditions (Figure 6).

Ascorbate increased the transcript level of *Rcd1* in wildtype and Rap2.4a knockout lines (Figure 6). In response to ascorbate, *2CPA* transcript levels only slightly changed in the RCD1 knock-out line *rcd1-3*, while they decreased in wildtype plants and increased in Rap2.4 knock-out lines. The fact that the 2CPA transcript amount is much less regulated by ascorbate in absence of RCD1 and that the response is inverted in absence of Rap2.4a demonstrates that RCD1 and Rap2.4a are essential for suppression of *2CPA* expression upon ascorbate application. However, unlike in Rap2.4a-dependent regulation (Shaikhali et al., 2008), there was no correlation between *RCD1* expression and *2CPA* transcript amount (Figure 6).

### RCD1 is an age-dependent regulator of the antioxidant system

Previously, we showed that the mRNA abundances of *Csd2, sAPx*, and *tAPx* are decreased in young *rcd1-6* plants in parallel to *2CPA* transcript levels (Heiber et al., 2007). To test the developmental stability of the RCD1 effect, the transcript abundance of genes encoding chloroplast proteins was analyzed in T19-2, *rcd1-3*, and in Rap2.4a knock-out lines in the rosette state.

**Figure 7:**
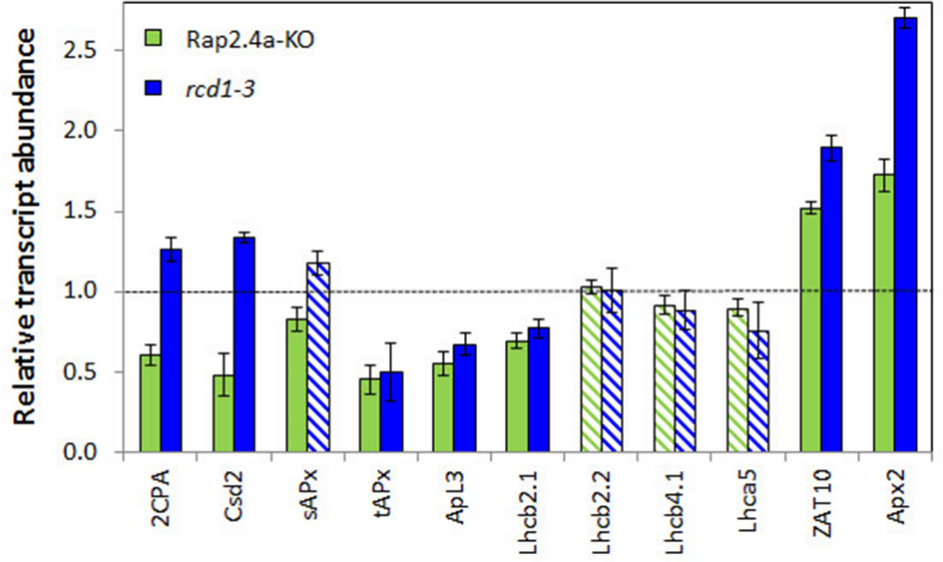
**Transcript levels of genes encoding chloroplast antioxidant enzymes (*2CPA, Csd2, sAPx*, and *tAPx*), other nuclear encoded chloroplast proteins (*APL3* and *LHCs*) and the stress inducible transcription factor *ZAT10* and its target gene *Apx2* in 6 week old Rap2.4a- and *rcd1-3* lines as determined by RT-PCR in actin standardized samples relative to wild-type plants**. The experiment was performed with three biological replicates and two technical replicates per independently grown biological replicate. Significant differences to the untreated control (Student's *T*-Test; α = 0.01; *n* = 3) are shown with filled bars, non-significant with striped bars.

At this stage, the 2CPA, Csd2, and sAPx transcript levels were slightly higher in the *rcd1*-mutant than in wildtype plants (Figure 7), demonstrating that the RCD1 control on these genes is lost during rosette development (Figure 7). The transcript levels were still dependent on Rap2.4a availability (Figure 7).

The transcript levels of *ApL3* (At4g39210; encoding the large subunit 3 of ADP glucose pyrophosphorylase) and the light harvesting complex protein *Lhcb2.1* (At3g61470) were still significantly decreased in 6 week old mutants. Expression of ROS-sensitive cytosolic ascorbate peroxidase 2 (*APx2*; At3g09640) and its inducing transcription factor *ZAT10* (At1g27730) (Karpinski et al., 1997; Mittler et al., 2006) was increased in the RCD-knock-out line (Figure 7), as reported before for *rcd1-6* seedlings (Heiber et al., 2007).

**Figure 8:**
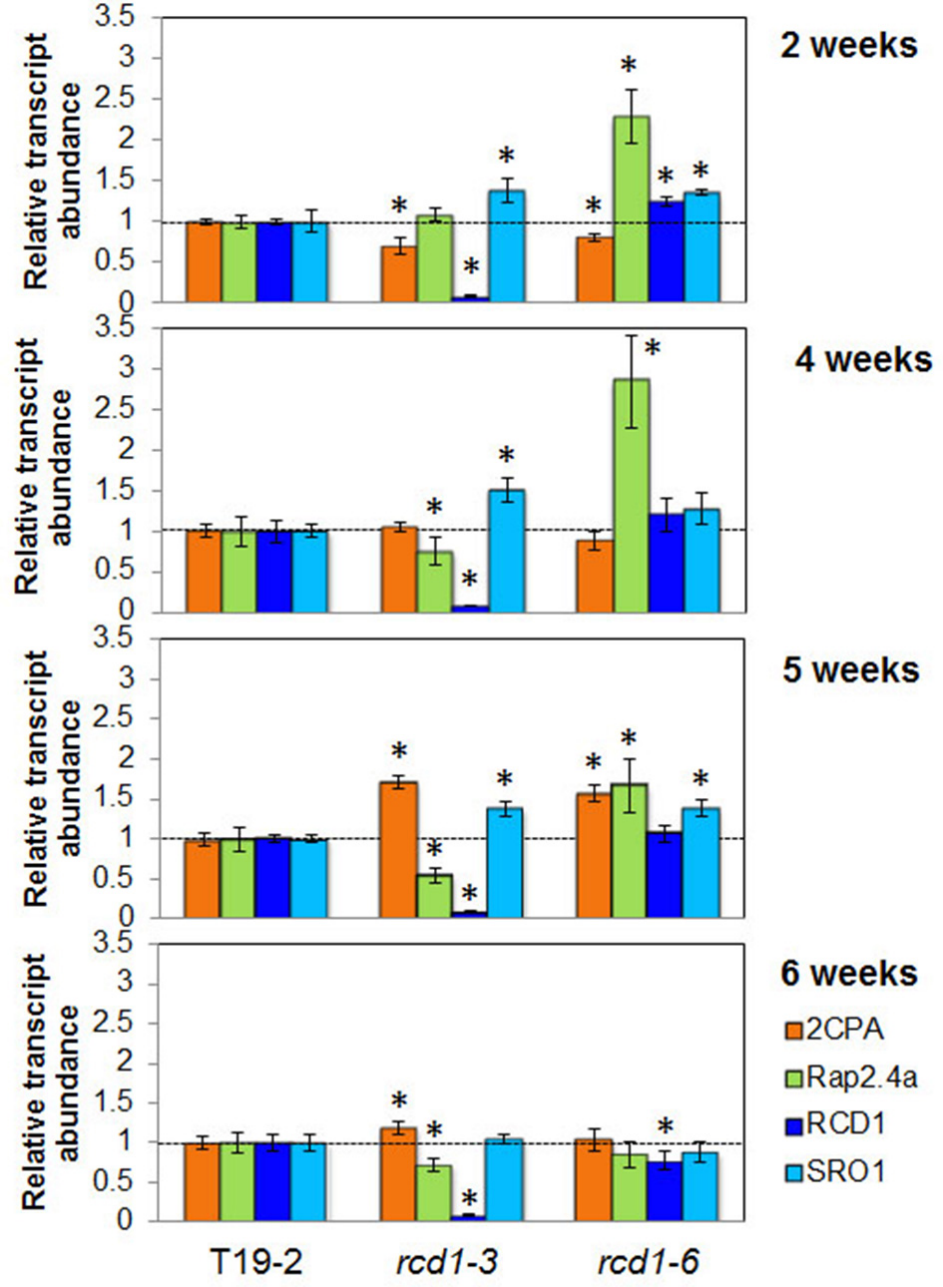
**Transcript levels of *2CPA, Rap2.4a, RCD1*, and *SRO1* in 2, 4, 5, and 6 week old Arabidopsis plants**. The transcript levels in T19-2, *rcd1-6* (*rimb1*), and the *rcd1-3* were determined by qRT-PCR for each sample in three biological replicates (pool of 5 plants) and two technical replicates and normalized on the transcript levels in the wildtype line. Significant differences (Student's *T*-Test; α = 0.01) are labeled with an asterisk. *RCD1* transcript levels were below the detection level in the T-DNA insertion line *rcd1-3*.

For more detailed analysis of the developmental regulation by RCD1, the transcript abundance regulation of *RCD1*, its closest homolog *SRO1, 2CPA*, and *Rap2.4a* was compared between 2, 4, 5, and 6 week old plants (Figure 8). The 2-Cys peroxiredoxin transcript levels were slighter decreased in young *rcd1-3* and *rcd1-6* plants consistent with previous data (Heiber et al., 2007; Figure 7). From 5 weeks onwards, when leaf formation and leaf elongation stopped, *2CPA* levels accumulated to higher levels in *rcd1-3* and *rcd1-6* than in the parental line T19-2 demonstrating that the RCD1 effect on *2CPA* expression is age-dependent and lost in mature tissues.

*RCD1* transcripts were barely detectable in the T-DNA insertion line *rcd1-3* (Teotia and Lamb, 2009; Figures 6, 8). In contrast, *rcd1-6*, which has a single nucleotide exchange in the coding region of the RCD1 gene, accumulated *RCD1*-mRNA to a slightly higher level than wildtype plants (T19-2) at 2 and 4 weeks age. Transcript abundance of *SRO1*, the closest homolog of *RCD1*, is similar in *rcd1-3* and *rcd1-6* with slightly more *SRO1* mRNA in *rcd1-3*. Compared to T19-2, the *SRO1* transcript levels were increased in both *rcd1* mutants in the first 5 weeks indicating a transient compensatory induction.

Surprisingly, the mutants *rcd1-3* and *rcd1-6* differed in the regulation of the *Rap2.4a* transcript amount. In *rcd1-6, Rap2.4a* transcript levels were increased to more than 150% at 2–5 weeks. In *rcd1-3 Rap2.4a* transcript levels were decreased in plants older than 2 weeks, indicating an allele-specific effect.

### Chloroplast ultrastructure

**Figure 9:**
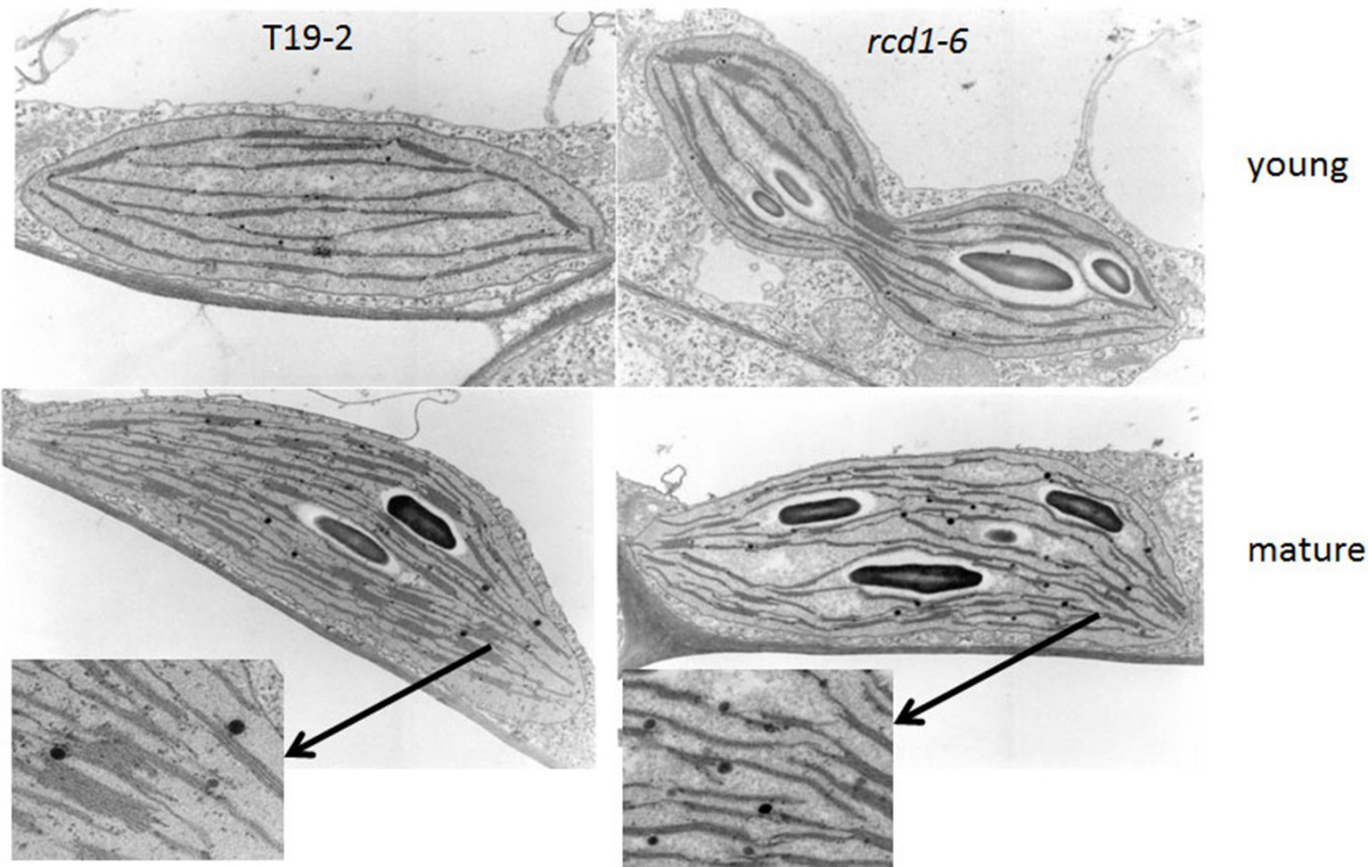
**Chloroplast ultrastructure of the *rcd1-6* mutant in comparison to its non-mutagenized parental line T19-2 in young leaves and in mature leaves**. Leaf sections were embedded, cut and analyzed. Representative chloroplasts are shown. From mature chloroplasts sections showing thylakoids are enlarged.

In the youngest leaves of 5 week old *rcd1-6* (*rimb1*) plants, chloroplasts were not significantly different from chloroplasts of the parental line T19-2, which were exposed to the same conditions, with respect of grana number and grana stacking intensity (Figure 9). In *rcd1-6*, starch granules were already observed at a very young developmental status, while the plastids were still dividing (Figure 9). Consistent with the slight chlorotic phenotype (Figures 1, 2; Heiber et al., 2007), the thylakoid stacking intensity was deceased to 2.36 ± 0.62 thylakoids in *rcd1-6* (*rimb1*) compared to 7.65 ± 1.82 thylakoids in T19-2 (Figure 9).

### Chlorophyll-a fluorescence analysis

**Figure 10:**
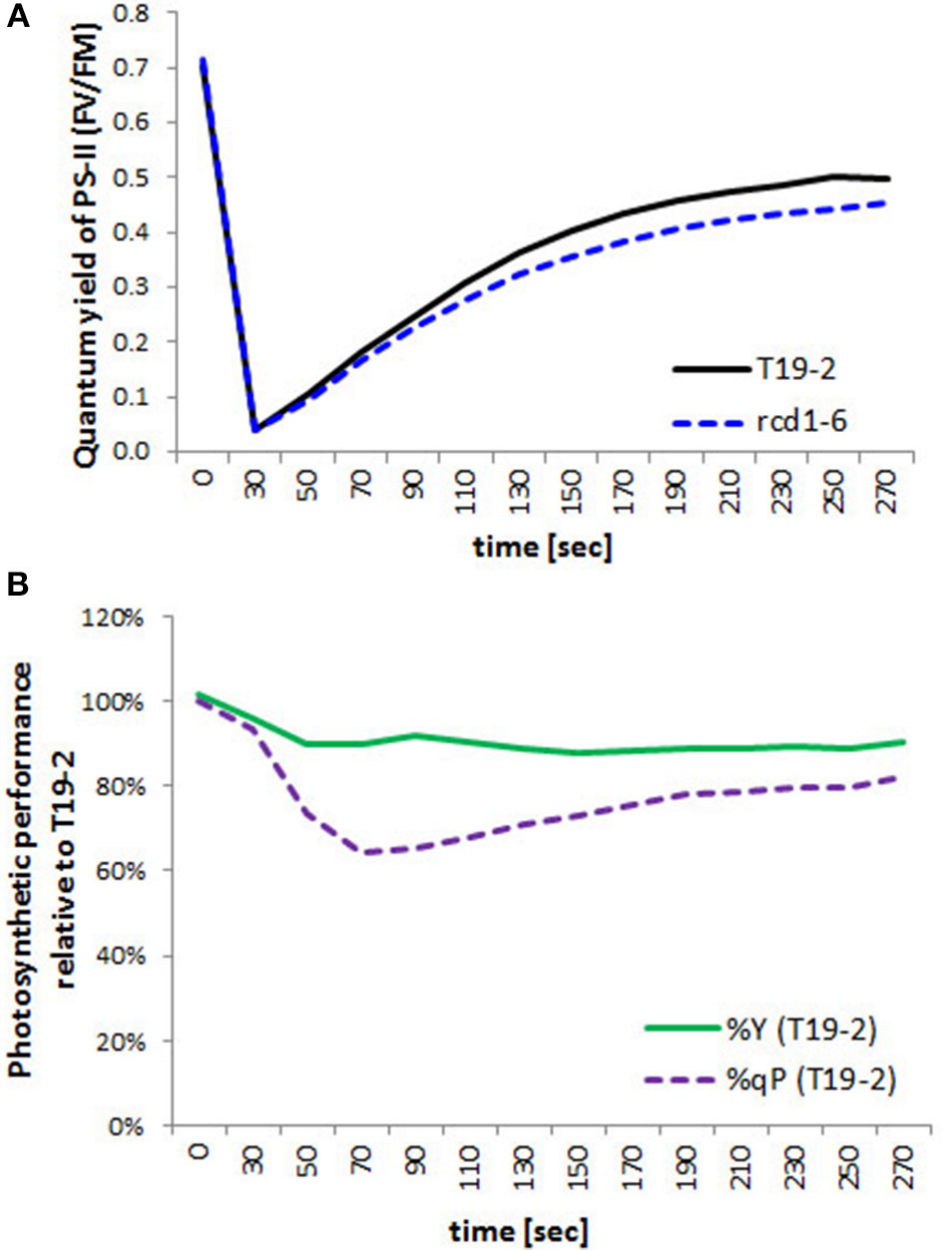
**Photosynthetic performance. (A)** Quantum Yield of photosystem I in *rcd1-6* (dotted line) in comparison to T19-2 (full line) after illumination of dark-adapted plant material. **(B)** Percentage of the quantum Yield (%Y; F_V_/F_M_; full line) and percentage of photochemical quenching (%qP; dotted line) of *rimb1* (*rcd1-6*) seedlings relative to T19-2 plants. Means were calculated from 10 samples of parallel grown 4 week old plants.

Chlorophyll-a fluorescence analysis enables a non-destructive look on chloroplast function (Schreiber and Bilger, 1993). The quantum yield of photosystem II reflects photosynthetic electron transport efficiency and photochemical quenching the oxidation of the plastoquinone pool and, therefore, under most conditions, activation of the Calvin-Benson-Cycle. Although the maximum quantum yield of photosystem II (F_V_/F_M_) (as determined immediately after 30 min dark relaxation) was similar in *rcd1-6 (rimb1)* and *T19-2* (Figure 10A), it increased slightly less in the light in the mutant. Between 45 s and 5 min of illumination, it was around 90% of that in T19-2 in *rimb1*.

Activation of photochemical quenching (qP) was much slower in *rcd1-6 (rimb1)* than in T19-2 for the first 90 s (Figure 10B). Ninety seconds after onset of light, it was only 62% that of T19-2 demonstrating problems with regulation of electron transfer within the photosynthetic electron transport chain or downstream.

## Discussion

### RCD1 regulates chloroplast antioxidant protection

The *rcd1-6* (*rimb1)* mutant was isolated for decreased transcriptional activity of the plant 2-Cys peroxiredoxin gene *2CPA* and decreased expression of other genes for chloroplast antioxidant enzymes (Heiber et al., 2007). The exchange of the codon for Trp-254 (TGG) into a stop codon (TAG) results in translation termination within the PARP-domain and before the RST-domain of the encoded protein in *rcd1-6* (Figure 1A). The C-terminal RST-domain is essential for interaction of RCD1 with transcription factors (Jaspers et al., 2009, 2010). RCD1 interacts with Rap2.4a in the complex protein environment of yeast cells (Figure 4), which regulates expression of various chloroplast antioxidant enzymes (Shaikhali et al., 2008).

RCD1 was first isolated in a screening approach aiming at the identification of plant homologs to the redox-regulated yeast transcription factor YAP1 (Belles-Boix et al., 2000). In yeast, YAP1 controls the expression of the oxidative stress regulon, including 2-Cys peroxiredoxin regulation (Lee et al., 1999). Lack of YAP1 function was complemented by RCD1 (Belles-Boix et al., 2000) due to its ability to form a scaffold for assembly of transcription factors (Jaspers et al., 2010). Mapping of *rimb1* to the RCD1 locus demonstrates that RCD1 promotes expression of the orthologous gene and therefore, conservation of (at least part of the) redox control pathways from yeast to plants.

*Rcd1-6* seedlings germinate and green normally (Heiber et al., 2007). Like in other *rcd1* alleles (Overmyer et al., 2005; Teotia and Lamb, 2009; Zhu et al., 2013), *rcd1* characteristic phenotypes develop in the rosette stage with maturation of the first true leaves. *rcd1-6* shows stunted growth, formation of aerial rosettes, decreased number of flowers and leaf damage as reported before for *rcd1-3* (Teotia and Lamb, 2009). Starting at the leaf rims, chlorosis and necrosis takes place. The leaves get serrated and turn yellow (Figures 1B, 2B). In addition, cells partially collapse within the mesophyll layer, which can be observed as clefts on the abaxial site of mature leaves (Figure 3B).

### Lesion formation results from insufficient chloroplast antioxidant protection

Lesion formation and hypersensitive responses correlate with ROS accumulation in *rcd1*-mutants (Overmyer et al., 2000; Zhu et al., 2013), which activates apoptotic processes (Foyer and Noctor, 2005; Baxter et al., 2014). In plants, programmed cell death is mainly triggered by chloroplasts in a combined action of photooxidative ROS production and signals correlating with a more reduced plastoquinone pool (Samuilov et al., 2003). RIMB1 was described as a major regulator in induction of the chloroplast antioxidant system (Heiber et al., 2007). Mapping of *rimb1* to the *rcd1* locus (Figure 1) linked two independently created lines of redox signaling analysis and provides novel insights into the function of RCD1:

In wildtype plants, the genes for chloroplast antioxidant enzymes are quickly and strongly activated at the onset of leaf development (Pena-Ahumada et al., 2006). This induction is limited in *rcd1-6* (Heiber et al., 2007). The redox buffering capacities are so low that in *rcd1-6* (*rimb1*) even slight changes in the light intensity, such as exposure to just 100 μmol quanta m^−2^ s^−1^ more than the plants were acclimated to, transiently result in a 38 % stronger reduction of the plastoquinone pool (1-qP; Figure 10) and the plants are very sensitive to variable environmental conditions (Suppl. 1).

Looking at much older plants (10–12 rosette leaves) than we routinely did (10 day old seedlings), Fujibe et al. (2004) showed higher ROS accumulation. Zhu et al. (2013) concluded that RCD1-typical induction of R-gene responses, activation of genes for extra-plastidic antioxidant enzymes and hypersensitive cell death result from the redox imbalances caused by insufficient antioxidant protection. Our work points out that the reason for insufficient antioxidant protection is caused early during plant development by limitations in the induction of genes for chloroplast antioxidant enzymes, while ROS-promoting greening and thylakoid development are not primarily affected.

One of the most puzzling results on RCD1 is that the *rcd1-6* mutant was isolated for its limitation in activation of the genes of the chloroplast antioxidant system and is, like other *rcd1* alleles (Ahlfors et al., 2004; Fujibe et al., 2004), more tolerant than its parental line to paraquat, which promotes photooxidative ROS production in chloroplasts (Mehler, 1951). In our opinion, paraquat tolerance of *rcd1* mutants is not linked in the first instance to the RCD1 function in the early activation of the chloroplast antioxidant system, but results from slower paraquat uptake due to differences in the epidermis structure, such as stress-induced stronger or denser cuticles upon stress (Dominguez et al., 2011) or the lower numbers of stomata (Figure 9). In this context it might be interesting to add that RCD1 also interacts with the AP2-type transcription factor DREB2A, which mediates drought responses (Vainonen et al., 2012).

The antioxidant system is almost at its limits in young wildtype Arabidopsis while it has to cope with developmental activation of photosynthesis (Pena-Ahumada et al., 2006). Limitations in the synthesis of enzymatic and non-enzymatic antioxidants further strain the chloroplast redox poise control in the *rcd1*-mutant (Heiber et al., 2007). As a consequence of insufficient protection, cells tend stronger to run into the cell death program (Figure 3B; Teotia and Lamb, 2011). During the time of these analyses, we moved twice our lab between universities. This gave us unintentionally the chance to see the mutant under different growth conditions. Comparison of plant populations grown over the time at various places, demonstrated that the extent of damage is conditional (Suppl. 1). Consistent with limitations in glutathione synthesis and the expression of genes for chloroplast antioxidant enzymes (Heiber et al., 2007) *rcd1* phenotypes are pronounced in a genetic background with low biosynthesis of the antioxidant ascorbate (Zhu et al., 2013). The lack of RCD1 caused also stronger phenotypes, when the mutants were exposed to ozone (Overmyer et al., 2005) or UV-B (Fujibe et al., 2004), or crossed with mutants limited in other stress response pathways, such as *snc1* (Zhu et al., 2013) and *ein2* (Overmyer et al., 2005).

### The RCD1 effect is age dependent

Overmyer et al. (2000) reported low expression of chloroplast Csd2 for *rcd1-1*, like we did for young *rcd1-6* (Heiber et al., 2007). The transcript level of the same gene was increased in the report of Fujibe et al. (2004) on *rcd1-2*. Between these experiments, the age of the plants and the growth conditions varied. Comparison of 2–6 week old plants demonstrated that the positive RCD1 effect on *2CPA, sAPx*, and *Csd2* expression can be inverted during development (Figure 8 in comparison to Heiber et al., 2007; Figure 9). RCD1 supports activation of the chloroplast antioxidant system specifically in young tissues. Besides RCD1-dependent regulation, additional stress-dependent activation can take place. As shown previously, for example higher light intensity (Heiber et al., 2007) and 23°C growth temperature (Fujibe et al., 2004), severe stress can induce expression of genes for chloroplast antioxidant enzymes in *rcd1* mutants in an RCD1-independent manner.

RCD1 and Rap2.4a regulation control preferentially genes for chloroplast antioxidant enzymes (Heiber et al., 2007; Shaikhali et al., 2008; Figure 7). They do not control the expression of genes for light-harvesting complex proteins or the also nuclear encoded small subunit of ribulose-1,5-bisphosphate carboxylase/oxygenase, which are also massively induced in young mesophyll cells in response to chloroplast signals (summarized in: Pfannschmidt, 2003; Baier and Dietz, 2005). Consistently, chlorophyll biosynthesis (Heiber et al., 2007) or early development of the chloroplast ultrastructure, such as thylakoid formation and initiation of grana stacking (Figure 9), take place similar as in wildtype plants.

### Position of RCD1 within the signaling network

RCD1 regulates, like Rap2.4a (Shaikhali et al., 2008), *sAPX, tAPx*, and *Csd2* expression (Heiber et al., 2007). Like Rap2.4a knockout lines (Shaikhali et al., 2008), *rcd1-mutants* can grow almost symptom-free (Overmyer et al., 2005; Heiber et al., 2007). Chlorosis, lesions and early leaf senescence preferentially develop in long-days or under fluctuating light conditions (Heiber et al., 2007; Shaikhali et al., 2008) (Suppl. 1). The physical interaction (Figure 4), the target overlap and the similar symptoms of knock-out lines (Heiber et al., 2007; Shaikhali et al., 2008; here) show that RCD1 functions as upstream-regulator in the Rap2.4a-mediated redox protection.

Positive interaction in yeast-two-hybrid tests (Figure 4) indicates that RCD1 acts as a direct upstream-regulators of Rap2.4a. Similar to the motif predicted by Vainonen et al. (2012) from the comparison of positive and negative interactions of RCD1 with AP2-type-transcription factors (FDXXELLXXLN) Rap2.4a has a FDXXEeaXXLa motif containing the essential FD-element and the E of the also experimentally proven ELL-motif (Vainonen et al., 2012).

Co-existence of RCD1 and Rap2.4a is essential for ascorbate-induced redox regulation of *2CPA* expression (Figure 6), while the interaction of Rap2.4a and RCD1 only slightly responds to oxidation and is insensitive to reduction in the yeast-two-hybrid assay (Figure 5). Regulation of the genes for chloroplast antioxidant enzymes takes place via a signal transduction pathway regulating Rap2.4a activity (Shaikhali et al., 2008; Shaikhali and Baier, 2010). The here presented data let assume that RCD1 only potentiates the Rap2.4a effect.

Rap2.4a availability supports *RCD1* expression (Figure 6) and RCD1 availability *Rap2.4a* expression (Figure 8). Truncated RCD1, which lacks the transcription factor binding site, is sufficient to increase the Rap2.4a transcript amount (Figure 8). Consequently, an auto-feedback loop depending on RCD1-Rap2.4 protein interaction can be excluded. Furthermore, *RCD1* overexpression does not overprotect, but gives a weak *rcd1* phenotype (Fujibe et al., 2006). We exclude direct feedback and suggest that Rap2.4a availability, which is essential for full expression of the genes for chloroplast antioxidant enzymes, is embedded in a transcription control network in which RCD1 masters the junctions.

### Interpretation of the root phenotype

Besides cell death in leaves, *rcd1* mutants show defects in root development (Teotia and Lamb, 2009) (Figure 3A). RCD1 and Rap2.4a are both strongly expressed in roots (Ahlfors et al., 2004; Shaikhali et al., 2008), where they may trigger a root-specific regulatory circuitry. Based on the data we collected for *rcd1-6* (*rimb1*) (Heiber et al., 2007 and here) we propose interpretation of the root defects as pleiotropic *root meristemless 1* (*rml1)*-like effect:

The *rml1* mutant is deficient in γ-glutamyl-cysteine synthase (Vernoux et al., 2000), which catalyzes the first step of glutathione synthesis. Due to lack of glutathione, root meristem function is lost very early in development and the plants die before post-embryonic shoot development is activated (Cheng et al., 1995). In *rcd1-6* (*rimb1*), the transcript levels of γ-glutamyl-cysteine synthase are only about half of the wildtype levels and the glutathione content is strongly decreased (Heiber et al., 2007). Due to also low enzymatic antioxidant support, the GSSG/GSH ratio is increased in *rcd1-6* (*rimb1*) (Heiber et al., 2007). In plants, γ-glutamyl-cysteine synthesis takes place in chloroplasts (Wachter et al., 2005), where C- and N-assimilation provide its educts. The limitations in activating the nuclear transcription of chloroplast γ-glutamyl-cysteine synthase gives *rcd1-6* a soft *rml1* mutant phenotype by disturbing redox-dependent cell-cycle control and, consequently, postembryonic root development and shoot meristematic activities (Vernoux et al., 2000; Teotia and Lamb, 2011).

The here described *rcd1-6* mutant shows weaker root elongation and branching effects than the *rcd1-3* allele described by Teotia and Lamb (2009). Since we grew our plants in short-days, while Teotia and Lamb (2009) worked under more straining long-day conditions (16 h, 80 μmol quanta m^−2^ s^−1^; 8 h darkness) (Becker et al., 2006; Queval et al., 2011) the phenotype difference fits with the general pattern of phenotype penetrance variation in *rcd1-6* (Suppl. 1).

## Conclusion

RCD1 was shown to control disease responses, cell death and the meristem fate in a ROS-dependent manner (Cheng et al., 1995; Overmyer et al., 2005; Teotia and Lamb, 2011). RIMB1 was characterized as a major regulator in the activation of chloroplast antioxidant system in seedlings and in the early rosette stage (Heiber et al., 2007). Mapping of *rimb1* to the *rcd1* locus and confirming the *rcd1*-phenotypes for *rimb1* linked the induction of disease response pathways and cell death to limited activation of the chloroplast antioxidant system.

With respect of signal transduction, we showed that RCD1 can interact with the transcription factor Rap2.4a, which was previously identified to activate various genes for chloroplast antioxidant enzymes and to be essential for protecting cells from photooxidative stress (Shaikhali et al., 2008). The RCD1-Rap2.4a interaction in yeast was barely redox-responsive. Together with the impact of RCD1 on Rap2.4a expression, it indicates that RCD1 does not perform, but support redox-regulation.

**Figure 11:**
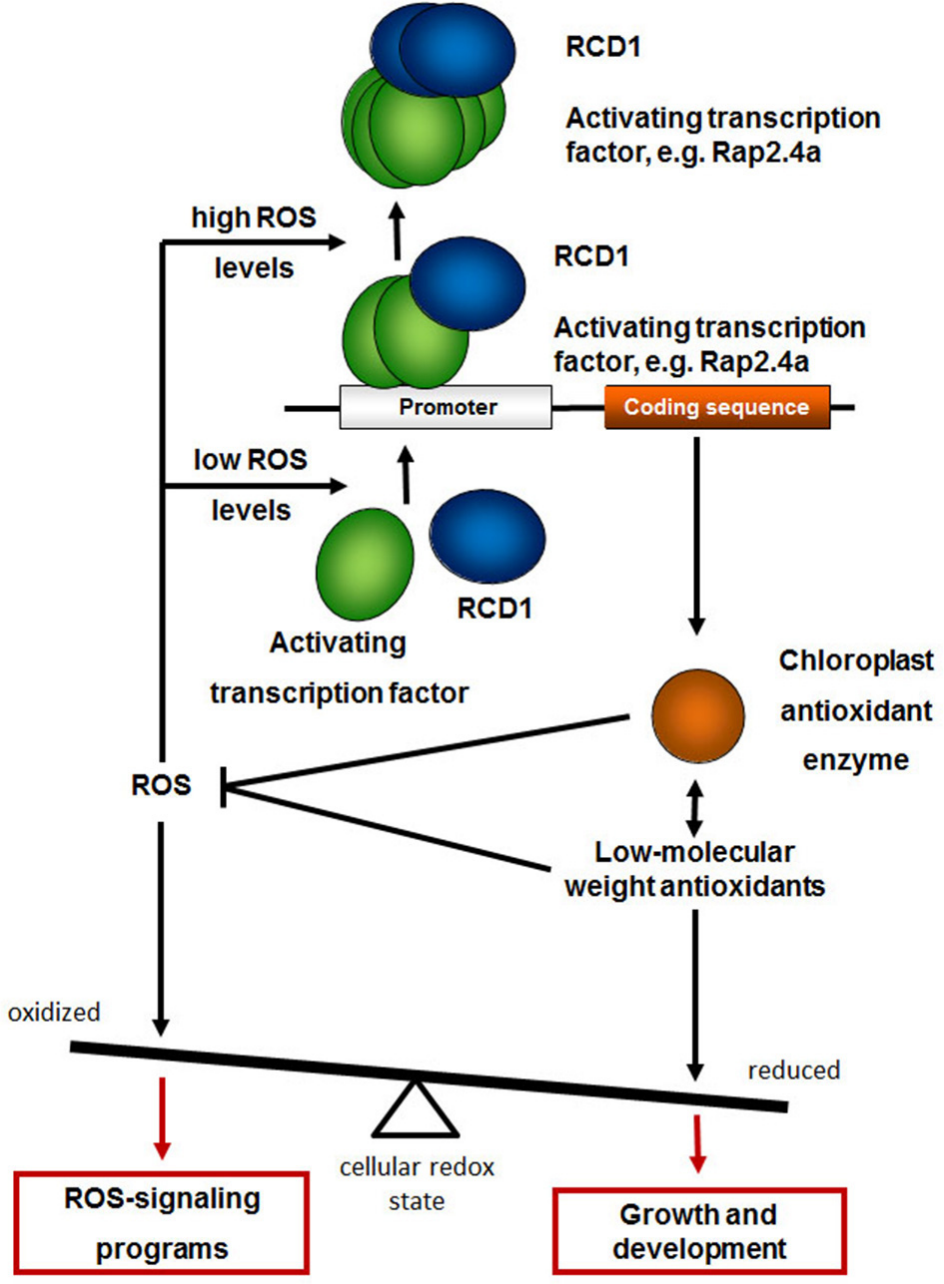
**RCD1 function in the regulation of genes for chloroplast antioxidant enzymes**. RCD1 promotes gene activation by the transcription factors Rap2.4a, which dimerizes upon slight redox imbalances and oligomerizes upon severe oxidative stress (Shaikhali et al., 2008). Expression of chloroplast antioxidant enzymes, such as 2CPA, and biosynthesis of low-molecular weight antioxidants keeps the cellular redox poise reduced and promotes growth and development. Upon insufficient antioxidant protection the cellular redox state gets more oxidized and activates ROS-signaling programs.

In *rcd1* mutants, ROS-sensitive signaling cascades, such as induction of *APx2* and *PR* genes (Miller et al., 2008), are activated (Heiber et al., 2007). Hypersensitive responses and cell death programs are promoted in absence of abiotic and biotic stressors (Teotia and Lamb, 2011; Zhu et al., 2013; Baxter et al., 2014). During development, the RCD1-impact on the activation of the chloroplast antioxidant system precedes the stress symptoms. In summary, we conclude that RCD1 protects plants cells from activation of ROS-signaling cascades by supporting regulation of genes encoding chloroplast antioxidant enzymes and glutathione biosynthesis (Figure 11).

## Accession numbers

*Actin*: At5g09810; *Act-2*: At3g18780; *ApL3*: At4g39210; *Apx2*: At3g09640, *2CPA*: At3g11630, *chlGR*: At3g54660; *Csd2*: At2g28190; *Lhca2.1*: At3g61470; *Lhca5*: At1g45474; *Lhcb2.2*: At2g05070; *Lhcb4.1*: At5g01530; *Rap2.4a*: At1g36060; *RCD1*: At1g32230; *sAP*x: At4g08390; *SRO1*: At2g35510; *tAPx*: At1g77490; *ZAT10*: At1g27730.

## Author contributions

Isabelle Heiber started the mapping population, Heiko Hiltscher mapped the *rimb1*-mutation and performed the comparative habitus studies. Jehad Shaikhali carried out the yeast-two-hybrid analysis and the ascorbate experiment. Radoslaw Rudnik, Marina Mellenthin, and Isabelle Heiber performed PCR-based transcript abundance analyses. Radoslaw Rudnik and Margarete Baier did the allelism tests. Günter Schuster and Heiko Hiltscher did the SEM studies, Iuri Meirelles Duarte and Heiko Hiltscher the light microscopy and Uwe Kahmann the TEM comparison of chloroplasts. Margarete Baier coordinated and supervised the project, performed the chlorophyll-a fluorescence analysis and drafted the manuscript based on sections contributed by Heiko Hiltscher, Isabelle Heiber, Jehad Shaikhali, and Radoslaw Rudnik and method descriptions by Günter Schuster.

### Conflict of interest statement

The authors declare that the research was conducted in the absence of any commercial or financial relationships that could be construed as a potential conflict of interest.

## Abbreviations

APx: ascorbate peroxidase
2-CP: 2-Cys peroxiredoxin
2CPA: 2-Cys peroxiredoxin A gene; aa: amino acid
AP2: apetala-2
3AT: 3-amino-1,2,4-triazol
BAC: Bacterial artificial chromosome
CAPS: cleaved amplified polymorphic sequences
Col-0: *Arabidopsis thaliana var*. *Columbia-0*
Csd: CuZn superoxide dismutase
DTT: dithiothreitol
F2/F3: filial generation 2/3
HR: hypersensitive response
Ler: *Arabidopsis thaliana var*. *Landsberg erecta*
PCD: programmed cell death
*rcd1*/RCD1: (gene encoding) Radical-induced cell death 1
*rimb1*: redox-imbalanced 1
ROS: reactive oxygen species
sAPx: stromal ascorbate peroxidase
SRO1: similar to RCD1
SSLP: single sequence length polymorphism
tAPx: thylakoid-bound ascorbate peroxidase
X-Gal: 5-Brom-4-chlor-3-indoxyl-β-D-galactopyranosid.

## References

[B1] Ahlfors R. Lang S. Overmyer K. Jaspers P. Brosche M. Tauriainen A. (2004). *Arabidopsis* RADICAL-INDUCED CELL DEATH1 belongs to the WWE protein-protein interaction domain protein family and modulates abscisic acid, ethylene, and methyl jasmonate responses. Plant Cell 16, 1925–1937 10.1105/tpc.021832 15208394 PMC514171

[B2] Armengaud P. Zambaux K. Hills A. Sulpice R. Pattison R. J. Blatt M. R. (2009). EZ-Rhizo: integrated software for the fast and accurate measurement of root system architecture. Plant J. 57, 945–956 10.1111/j.1365-313X.2008.03739.x 19000163

[B3] Arvidsson S. Kwasniewski M. Riano-Pachon D. M. Mueller-Roeber B. (2008). QuantPrime - a flexible tool for reliable high-throughput primer design for quantitative PCR. BMC Bioinformatics 9:465 10.1186/1471-2105-9-465 18976492 PMC2612009

[B4] Ausubel F. M. Berndt K. D. Holmgren A. (2001). Current Protocol in Molecular Biology. New York, NY: Wiley 10.1002/0471142727

[B5] Baier M. Dietz K.-J. (1999). The costs and benefits of oxygen for photosynthesizing plant cells. Prog. Bot. 60, 282–314 10.1007/978-3-642-59940-8_11

[B6] Baier M. Dietz K.-J. (2005). Chloroplasts as source and target of cellular redox regulation: a discussion of chloroplast-to-nucleus signals in context of plant physiology and evolution. J. Exp. Bot. 56, 1449–1462 10.1093/jxb/eri161 15863449

[B7] Baier M. Noctor G. Foyer C. H. Dietz K. J. (2000). Antisense suppression of 2-cysteine peroxiredoxin in *Arabidopsis* specifically enhances the activities and expression of enzymes associated with ascorbate metabolism but not glutathione metabolism. Plant Physiol. 124, 823–832 10.1104/pp.124.2.823 11027730 PMC59186

[B8] Baier M. Ströher E. Dietz K. J. (2004). The acceptor availability at photosystem I and ABA control nuclear expression of 2-Cys peroxiredoxin-A in *Arabidopsis thaliana*. Plant Cell Physiol. 45, 997–1006 10.1093/pcp/pch114 15356325

[B9] Baxter A. Mittler R. Suzuki N. (2014). ROS as key players in plant stress signalling. J. Exp. Bot. 65, 1229–1240 10.1093/jxb/ert375 24253197

[B10] Bechtold U. Richard O. Zamboni A. Gapper C. Geisler M. Pogson B. (2008). Impact of chloroplastic- and extracellular-sourced ROS on high light-responsive gene expression in Arabidopsis. J. Exp. Bot. 59, 121–133 10.1093/jxb/erm289 18212028

[B11] Becker B. Holtgrefe S. Jung S. Wunrau C. Kandlbinder A. Baier M. (2006). Influence of the photoperiod on redox regulation and stress responses in *Arabidopsis thaliana* L. (Heynh.) plants under long- and short-day conditions Planta 224, 380–393 10.1007/s00425-006-0222-3 16435132

[B12] Belles-Boix E. Babiychuk E. Van Montagu M. Inze D. Kushnir S. (2000). CEO1, a new protein from *Arabidopsis thaliana*, protects yeast against oxidative damage. FEBS Lett. 482, 19–24 10.1016/S0014-5793(00)02016-0 11018516

[B13] Cheng J.-C. Seeley K. A. Sung Z. R. (1995). RML1 and RML2, *Arabidopsis* genes required for cell proliferation at the root tip. Plant Physiol. 107, 365–376 10.1104/pp.107.2.365 7724670 PMC157136

[B14] Dominguez E. Heredia-Guerrero J. A. Heredia A. (2011). The biophysical design of plant cuticles: an overview. New Phytol. 189, 938–949 10.1111/j.1469-8137.2010.03553.x 21374891

[B15] Foyer C. H. Lelandais M. Kunert K. J. (1994). Photooxidative stress in plants. Physiol. Plant. 92, 696–717 10.1111/j.1399-3054.1994.tb03042.x

[B16] Foyer C. H. Noctor G. (2005). Redox homeostasis and antioxidant signaling: a metabolic interface between stress perception and physiological responses. Plant Cell 17, 1866–1875 10.1105/tpc.105.033589 15987996 PMC1167537

[B17] Fujibe T. Saji H. Arakawa K. Yabe N. Takeuchi Y. Yamamoto K. T. (2004). A methyl viologen-resistant mutant of Arabidopsis, which is allelic to ozone-sensitive *rcd1*, is tolerant to supplemental ultraviolet-B irradiation. Plant Physiol. 134, 275–285 10.1104/pp.103.033480 14657410 PMC316307

[B17a] Fujibe T. Saji H. Watahiki M. K. Yamamoto K. T. (2006). Overexpression of the Radical-induced Cell Death 1 (Rcd1) Gene of Arabidopsis causes weak RCD1 phenotype with compromised oxidative-stress responses. Biosci. Biotechnol. Biochem. 70, 1827–1831 16926493 10.1271/bbb.50673

[B18] Heiber I. Ströher E. Raatz B. Busse I. Kahmann U. Bevan M. W. (2007). The redox imbalanced mutants of *Arabidopsis* differentiate signaling pathways for redox regulation of chloroplast antioxidant enzymes. Plant Physiol. 143, 1774–1788 10.1104/pp.106.093328 17337533 PMC1851819

[B19] Horling F. Lamkemeyer P. König J. Finkemeier I. Kandlbinder A. Baier M. (2003). Divergent light-, ascorbate-, and oxidative stress-dependent regulation of expression of the peroxiredoxin gene family in *Arabidopsis*. Plant Physiol. 131, 317–325 10.1104/pp.010017 12529539 PMC166811

[B20] Jander G. Norris S. R. Rounsley S. D. Bush D. F. Levin I. M. Last R. L. (2002). *Arabidopsis* map-based cloning in the post-genome era. Plant Physiol 129, 440–450 10.1104/pp.003533 12068090 PMC1540230

[B21a] Jaspers P. Blomster T. Brosché M. Salojärvi J. Ahlfors R. Vainonen J. P. (2009). Unequally redundant RCD1 and SRO1 mediate stress and developmental responses and interact with transcription factors. Plant J. 60, 268–279 10.1111/j.1365-313X.2009.03951.x 19548978

[B21] Jaspers P. Brosche M. Overmyer K. Kangasjärvi J. (2010). The transcription factor interacting protein RCD1 contains a novel conserved domain. Plant Signal. Behav. 5, 78–80 10.4161/psb.5.1.10293 20592818 PMC2835967

[B23] Juszczak I. Rudnik R. Pietzenuk B. Baier M. (2012). Natural genetic variation in the expression regulation of the chloroplast antioxidant system among *Arabidopsis thaliana* accessions. Physiol. Plant. 146, 53–70 10.1111/j.1399-3054.2012.01602.x 22339086

[B24] Kangasjärvi S. Lepisto A. Hannikainen K. Piippo M. Luomala E. M. Aro E. M. (2008). Diverse roles for chloroplast stromal and thylakoid-bound ascorbate peroxidases in plant stress responses. Biochem. J. 412, 275–285 10.1042/BJ20080030 18318659

[B25] Karpinski S. Escobar C. Karpinska B. Creissen G. Mullineaux P. M. (1997). Photosynthetic electron transport regulates the expression of cytosolic ascorbate peroxidase genes in *Arabidopsis* excess light stress. Plant Cell 9, 627–640 10.1105/tpc.9.4.627 9144965 PMC156944

[B26] Katiyar-Agarwal S. Zhu J. Kim K. Agarwal M. Fu X. Huang A. (2006). The plasma membrane Na+/H+ antiporter SOS1 interacts with RCD1 and functions in oxidative stress tolerance in Arabidopsis. Proc. Natl. Acad. Sci. U.S.A. 103, 18816–18821 10.1073/pnas.0604711103 17023541 PMC1693745

[B27] König J. Baier M. Horling F. Kahmann U. Harris G. Schürmann P. (2002). The plant-specific function of 2-Cys peroxiredoxin-mediated detoxification of peroxides in the redox-hierarchy of photosynthetic electron flux. Proc. Natl. Acad. Sci. U.S.A. 99, 5738–5743 10.1073/pnas.072644999 11929977 PMC122841

[B28] Lee J. Godon C. Lagniel G. Spector D. Garin J. Labarre J. (1999). Yap1 and Skn1 control specialized oxidative stress response regulons in yeast. J. Biol. Chem. 274, 16040–16046 10.1074/jbc.274.23.16040 10347154

[B29] Li B. Fields S. (1993). Identification of mutations in p53 that affect its binding to SV40 large T antigen by using the yeast-two-hybrid system. FASEB J. 7, 957–963 8344494 10.1096/fasebj.7.10.8344494

[B30] Li L. Elledge S. J. Peterson C. A. Bales E. S. Legerski R. J. (1994). Specific association between the human Dna-Repair proteins Xpa and Ercc1. Proc. Natl. Acad. Sci. U.S.A. 91, 5012–5016 10.1073/pnas.91.11.5012 8197174 PMC43920

[B31] Mehler A. H. (1951). Studies on reactions of illuminated chloroplasts. II. Stimulation and inhibition of reactions with molecular oxygen. Arch. Biochem. Biophys. 33, 339–351 10.1016/0003-9861(51)90012-4 14904069

[B32] Miller G. Shulaev V. Mittler R. (2008). Reactive oxygen signaling and abiotic stress. Physiol. Plant. 133, 481–489 10.1111/j.1399-3054.2008.01090.x 18346071

[B33] Mittler R. Kim Y. Song L. H. Coutu J. Coutu A. Ciftci-Yilmaz S. (2006). Gain- and loss-of-function mutations in Zat10 enhance the tolerance of plants to abiotic stress. Febs Lett. 580, 6537–6542 10.1016/j.febslet.2006.11.002 17112521 PMC1773020

[B34] Overmyer K. Brosche M. Pellinen R. Kuittinen T. Tuominen H. Ahlfors R. (2005). Ozone-induced programmed cell death in the *Arabidopsis* radical-induced cell death1 mutant. Plant Physiol. 137, 1092–1104 10.1104/pp.104.055681 15728341 PMC1065409

[B34a] Overmyer L. Tuominen H. Kettunen R. Betz C. Langebartels C. Sandermann H. (2000). Ozone-sensitive Arabidopsis rcd1 mutant reveals opposite roles for ethylene and jasmonate signaling pathways in regulating superoxide-dependent cell death. Plant Cell 12, 1849–1862 10.1105/tpc.12.10.1849 11041881 PMC149124

[B35] Pena-Ahumada A. Kahmann U. Dietz K. J. Baier M. (2006). Regulation of peroxiredoxin expression versus expression of Halliwell-Asada-Cycle enzymes during early seedling development of *Arabidopsis thaliana*. Photosynth. Res. 89, 99–112 10.1007/s11120-006-9087-3 16915352

[B36] Pfannschmidt T. (2003). Chloroplast redox signals: how photosynthesis controls its own genes. Trends Plant Sci. 8, 33–41 10.1016/S1360-1385(02)00005-5 12523998

[B37] Queval G. Jaillard D. Zechmann B. Noctor G. (2011). Increased intracellular H_2_O_2_ availability preferentially drives glutathione accumulation in vacuoles and chloroplasts. Plant Cell Environ. 34, 21–32 10.1111/j.1365-3040.2010.02222.x 20807372

[B22] Rossel J. B. Wilson P. B. Hussain D. Woo N. S. Gordon M. J. Mewett O. P. (2007). Systemic and intracellular responses to photooxidative stress in *Arabidopsis*. Plant Cell 19, 4091–4110 10.1105/tpc.106.045898 18156220 PMC2217654

[B38a] Samuilov V. D. Lagunova E. M. Dzyubinskaya E. V. Izyumov D. S. Kiselevsky D. B. Makarova Y. V. (2002). Involvement of chloroplasts in the programmed cell death of plant cells. Biochemistry 67, 627–634 10.1023/A:1016138003183 12126469

[B38] Samuilov V. D. Lagunova E. M. Kiselevsky D. B. Dzyubinskaya E. V. Makarova Y. V. Gusev M. V. (2003). Participation of chloroplasts in plant apoptosis. Biosci. Rep. 23, 103–117 10.1023/A:1025576307912 14570380

[B39] Schneider S. Buchert M. Hovens C. M. (1996). An *in vitro* assay of beta-galactosidase from yeast. Biotechniques 20, 960–962 8780862 10.2144/96206bm03

[B40] Schreiber U. Bilger W. (1993). Progress in chlorophyll fluorescence research: major developments during the past years in retrospect. Prog. Bot. 54, 151–173

[B41] Shaikhali J. Baier M. (2010). Ascorbate regulation of 2-Cys peroxiredoxin-A promoter activity is light-dependent. J. Plant Physiol. 167, 461–467 10.1016/j.jplph.2009.10.021 20022402

[B42] Shaikhali J. Heiber I. Seidel T. Ströher E. Hiltscher H. Birkmann S. (2008). The redox-sensitive transcription factor Rap2.4a controls nuclear expression of 2-Cys peroxiredoxin A and other chloroplast antioxidant enzymes. BMC Plant Biol. 8:48 10.1186/1471-2229-8-48 18439303 PMC2386467

[B43] Sheffield J. B. (2007). ImageJ, a useful tool for biological image processing and analysis. Microsc. Microanal. 13, 200–201 10.1017/S1431927607076611

[B44] Teotia S. Lamb R. S. (2009). The paralogous genes RADICAL-INDUCED CELL DEATH1 and SIMILAR TO RCD ONE1 have partially redundant functions during *Arabidopsis* development. Plant Physiol. 151, 180–198 10.1104/pp.109.142786 19625634 PMC2736012

[B45] Teotia S. Lamb R. S. (2011). RCD1 and SRO1 are necessary to maintain meristematic fate in *Arabidopsis thaliana*. J. Exp. Bot. 62, 1271–1284 10.1093/jxb/erq363 21172813 PMC3022410

[B46] Vainonen J. P. Jaspers P. Wrzaczek M. Lamminmaki A. Reddy R. A. Vaahtera L. (2012). RCD1-DREB2A interaction in leaf senescence and stress responses in *Arabidopsis thaliana*. Biochem. J. 442, 573–581 10.1042/BJ20111739 22150398

[B47] Vernoux T. Wilson R. C. Seeley K. A. Reichheld J. P. Muroy S. Brown S. (2000). The ROOT MERISTEMLESS1/CADMIUM SENSITIVE2 gene defines a glutathione-dependent pathway involved in initiation and maintenance of cell division during postembryonic root development. Plant Cell 12, 97–110 10.1105/tpc.12.1.97 10634910 PMC140217

[B48] Wachter A. Wolf S. Steininger H. Bogs J. Rausch T. (2005). Differential targeting of GSH1 and GSH2 is achieved by multiple transcription initiation: implications for the compartmentation of glutathione biosynthesis in the Brassicaceae. Plant J. 41, 15–30 10.1111/j.1365-313X.2004.02269.x 15610346

[B49] Wood Z. A. Poole L. B. Karplus P. A. (2003). Peroxiredoxin evolution and the regulation of hydrogen peroxide signaling. Science 300, 650–653 10.1126/science.1080405 12714747

[B50] Zapata J. M. Guera A. Esteban-Carrasco A. Martin M. Sabater B. (2005). Chloroplasts regulate leaf senescence: delayed senescence in transgenic ndhF-defective tobacco. Cell Death Differ. 12, 1277–1284 10.1038/sj.cdd.4401657 15905880

[B51] Zhu Y. Du B. Qian J. Zou B. Hua J. (2013). Disease resistance gene-induced growth inhibition is enhanced by rcd1 independent of defense activation in *Arabidopsis*. Plant Physiol. 161, 2005–2013 10.1104/pp.112.213363 23365132 PMC3613471

