# Supplementary material for: The radical induced cell death protein 1 (RCD1) supports transcriptional activation of genes for chloroplast antioxidant enzymes
Source: Front Plant Sci. 2014 Sep 23;5:475. doi: 10.3389/fpls.2014.00475 (PMC4172000; doi:10.3389/fpls.2014.00475)
Supplement: Supplementary file 1 [file DataSheet1.PDF]

Supplements

Suppl.1: Penetrance test. Independent plant sets of 92 – 412 plants were grown in the growth chamber or in the greenhouse.

Abnormal rosette phenotype	***T19-2***	***rcd1-6***
Bielefeld – growth chamber (short day)	4.3 %	69.4 %
Bielefeld – greenhouse early March (short day)	2.1%	27.3%
Bielefeld – greenhouse late March (almost equal days and nights)	8.3 %	74.2 %
Bielefeld - greenhouse June (long day)	8.7 %	89.5%
Düsseldorf – growth chamber (short day)	3.7%	49.8%
Düsseldorf – growth chamber (short day)	3.2%	53.8%
Düsseldorf – growth chamber (short day; Bielefeld soil type)	5.1%	63.6%
Düsseldorf – greenhouse March (long day, temperature control)	5.8 %	68.2%
Düsseldorf – greenhouse June (long day, no temperature control)	12.3 %	98.4%
Berlin set 1 – growth chamber (short day)	1.7%	57.3%
Berlin set 2 – growth chamber (short day)	1.3 %	62.1%
Berlin set 3 – growth chamber (short day, soft drought stress)	2.7 %	79.8 %
Berlin set 4 – growth chamber (short day, 260 µmol quanta m^{-2} s^{-1})	1.9 %	56.9 %

Suppl. 2: 5´-UTR-linked primers for RT-PCR

Gene	Gene code	Forward primer<br>Reverse primer	Annealing temperature
Actin	At5g09810	GAGAAGATGACTCAGATC<br>ATCCTTCCTGATATCGACATCA	52
2CPA	At3g11630	CTCTCCATCTGTTTCTTT<br>GTACCTTTTTGCTATCAT	52
Csd2	At2g28190	CTCCGTTCCTCTTTCAGC<br>GCGTCAAGCCAATCACAC	52
sAPx	At4g08390	TGTTCCAGTTAGCTAGTG<br>GGTTGAGTAAATTAGGTGC	52
tAPx	At1g77490	AATAGTTGCCTTGTCTGG<br>GGAATATATGATCACCACG	52
APL3	At4g39210	CGATCTGAAATCAATGCAAACC<br>GCATTTCCTGATCTTTGTATCTCG	52
Lhca2.1	At3g61470	CTCAAAGCATTTGGTACG<br>TTAGTAGCGTAAGACCAG	52
Lhca5	At1g45474	GGAGATTACGGGTTTGAC<br>TGATGGTCTTATGCCATG	52
Lhcb2.2	At2g05070	CGTCTTACTTAACTGGAG<br>TTAGTAGCGTAAGACCAG	52
Lhcb4.1	At5g01530	CGGAATCCAGAGATTCAG<br>CCCAATTGTTGAGTGGAC	52
ZAT10	At1g27730	CTCTTACATCACCAAGATTAG<br>TCAAACCGAGGCTTCTTCG	56
Apx2	At3g09640	ACCCGCTCATTTTTGACAAC<br>AGCAAACCCGAGTTCTGACA	56
RCD1	At1g32230	CGATGACATTGAGAGTCC<br>CTGATGCTTCCTGGAATCTC	55
Rap2.4a	At1g36060	ATGGCGGATCTCTTCGGTG<br>GTCCTGTCTATTCATCAGTCG	55

Suppl. 3: Exon-intron-border spanning primers for qRT-PCR

Gene	Gene code	Forward primer<br>Reverse primer	Annealing temperature
Act2	At3g18780	AATCACAGCACTTGCACCAAGC<br>CCTTGGAGATCCACATCTGCTG	60
2CPA	At3g11630	CCCAACAGAGATTACTGCCT<br>ATAGTTCAGATCACCAAGCCC	60
RCD1	At1g32230	CCTCCTCAATTGGAGTCAAACCAG<br>GGGTCTTGTAGTGCTTGAACCAAC	60
SRO1	At2g35510	CTGCAAACTGCCCTTATTTCAGTG<br>GCGTGTTATCACCACGAAGAGG	60
Rap2.4a	At1g36060	CGTCAGCGCCACAACAACATTC<br>AACATCCGAAGTCGGTGAACCC	60
RCD1	At1g32230	CCTCCTCAATTGGAGTCAAACCAG<br>GGGTCTTGTAGTGCTTGAACCAAC	60
SRO1	At2g35510	CTGCAAACTGCCCTTATTTCAGTG<br>GCGTGTTATCACCACGAAGAGG	60
